# Measurement Invariance in Longitudinal Bifactor Models: Review and Application Based on the *p* Factor

**DOI:** 10.1177/10731911231182687

**Published:** 2023-06-22

**Authors:** Sharon A. S. Neufeld, Michelle St Clair, Jeannette Brodbeck, Paul O. Wilkinson, Ian M. Goodyer, Peter B. Jones

**Affiliations:** 1 University of Cambridge, UK; 2 University of Bath, UK; 3 University of Bern, Switzerland

**Keywords:** longitudinal bifactor modeling, measurement invariance, review, simulation studies, *p* factor (general psychopathology)

## Abstract

Bifactor models are increasingly being utilized to study latent constructs such as psychopathology and cognition, which change over the lifespan. Although longitudinal measurement invariance (MI) testing helps ensure valid interpretation of change in a construct over time, this is rarely and inconsistently performed in bifactor models. Our review of MI simulation literature revealed that only one study assessed MI in bifactor models under limited conditions. Recommendations for how to assess MI in bifactor models are suggested based on existing simulation studies of related models. Estimator choice and influence of missing data on MI are also discussed. An empirical example based on a model of the general psychopathology factor (*p*) elucidates our recommendations, with the present model of *p* being the first to exhibit residual MI across gender and time. Thus, changes in the ordered-categorical indicators can be attributed to changes in the latent factors. However, further work is needed to clarify MI guidelines for bifactor models, including considering the impact of model complexity and number of indicators. Nonetheless, using the guidelines justified herein to establish MI allows findings from bifactor models to be more confidently interpreted, increasing their comparability and utility.

Bifactor models are increasingly being used to model multidimensional constructs such as psychopathology and cognition to generate distinct uncorrelated factors containing shared variance common across all model indicators (“general factor”) and variance shared by only a subset of indicators (“specific factors”) (Markon, 2019). The factor orthogonality in these confirmatory factor models contrasts with the more constrained higher-order model, where specific factors are nested in the general factor (Markon, 2019). This orthogonality suggests that the bifactor model can be used to discern unique effects simultaneously across factors (Lahey et al., 2021), although this poses its own challenges (Markon, 2019). Caution is needed when interpreting bifactor models, as they tend to overfit data and thus should not be adjudicated by fit statistics alone (Bonifay & Cai, 2017). Factor reliabilities tend to be strong for the general factor, but far less consistent for specific factors, calling into question their interpretation (Rodriguez et al., 2016; Watts et al., 2020). Nonetheless, bifactor models are commonly applied to estimate constructs that are known to undergo change over the lifespan (Caspi & Moffitt, 2018; Deary, 2012; Markon, 2019). When modeling any latent construct over time, longitudinal measurement invariance (MI) should be established to ensure that observed changes reflect genuine differences in the construct over time and not in the measurement model (van de Schoot et al., 2012). However, to date there have been very few studies that have assessed longitudinal MI in bifactor models. Our literature review (Figure 1) revealed only 10 articles across all subject areas that assessed longitudinal MI in a confirmatory a bifactor model. In these studies, there was inconsistency in what level MI was tested to, and which fit indices and cut-offs were used for determining MI. We postulate this is in large part due to inadequate guidelines for MI testing in bifactor models; thus, a review of the existing evidence is warranted.

**Figure 1.**
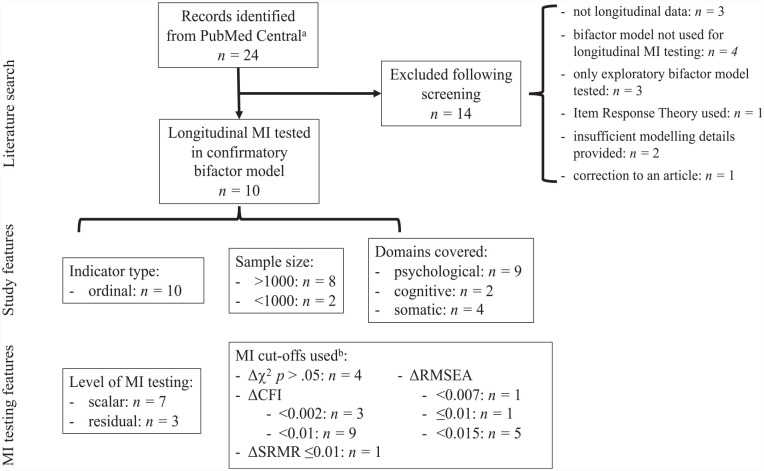
Literature Search of Articles Indicating Longitudinal MI Testing, and the MI Features of Identified Articles. *Note.* MI = measurement invariance; Δχ^2^ = chi-square difference test; CFI = comparative fit index; RMSEA = root mean square error of approximation; SRMR = standardized root mean squared residual. ^a^Searched on February 22, 2023: (bifactor[All Fields] OR bi-factor[All Fields]) AND (measurement invariance[All Fields] OR measurement equivalence[All Fields] or invarian*[All Fields]) AND (“longitudinal*”[All Fields] OR “time-series”[All Fields] OR “prospectiv*”[All Fields] OR “follow-up”[All Fields]). Deutz et al. (2016) were not identified in this literature search but are included in the text when these 10 studies are mentioned, as all other *p*-factor studies that demonstrated longitudinal scalar MI were found using the above search terms. ^b^ All but one study used multiple indices or cut-offs.

This study is organized as follows. Section “MI Testing in Bifactor Models” focuses on longitudinal MI testing in bifactor models. First, we outline the methods of MI testing. Second, to devise practical guidelines for assessing MI in bifactor models, we review existing MI cut-offs from simulation studies under a variety of conditions. Third, estimator choices and influence of missing data on MI are reviewed. Fourth, we review the literature on longitudinal bifactor models of psychopathology as a case study to assess how well this literature has adhered to the above MI testing guidelines. Section “Empirical Example” provides an empirical example testing MI in a bifactor model of psychopathology specified by ordered-categorical indicators; section “General Discussion” concludes with a general discussion.

## MI Testing in Bifactor Models

### MI Testing Methodology

MI is typically tested using multiple group confirmatory factor analysis (MG-CFA) with groups (e.g., gender) defined in a between-subjects manner (Vandenberg & Lance, 2000). Longitudinal MI is often performed in a single-group CFA (with wide formatted data), to account for the dependent nature of the data (Vandenberg & Lance, 2000). A related approach to testing MI in ordered-categorical data, item response theory (IRT), requires testing each item individually for differential item functioning (DIF) (D’Urso et al., 2021). This is less practical for large models than CFA-based MI testing, where MI is tested for all items at once. Consequently, our literature review of longitudinal MI testing in bifactor models only revealed one study where IRT was used (Figure 1), and only CFA was used for MI testing in the bifactor models of psychopathology we review below. Furthermore, simulations have shown that compared with IRT, scale-level MG-CFA more correctly identifies non-invariance in ordered-categorical items (D’Urso et al., 2021). Thus, the focus of the present article is on CFA approaches to MI testing, with parallels drawn with IRT where relevant.

A forward hierarchical approach to MI testing using nested models has shown greater accuracy in detecting non-invariance than starting with the presumption of invariance (Stark et al., 2006). Starting with a minimally constrained model, invariance is supported if the model fit does not substantially deteriorate following additional constraints. The method for *comparing fit between nested models* is a subject of some debate (Putnick & Bornstein, 2016) and will be addressed more fully below.

The steps for MI testing are as follows. If the model fits well in each group/timepoint (or fits well in a multigroup or multiwave model without equity constraints), this suggests that the number of factors and patterns of loadings are equivalent in each group/timepoint and *configural invariance* is established (van de Schoot et al., 2012). If configural invariance is not established, this indicates that the factor structure is not consistent across groups/timepoints and MI testing is stopped. Following demonstration of configural invariance, factor loadings can then be fixed to equity across groups/timepoints to test for *metric (or weak factorial) invariance*. Establishing metric invariance with continuous indicators implies that the respondents attribute the same meaning to the construct over groups/timepoints (van de Schoot et al., 2012). Thus, the variances (and cross-wave covariances) of the latent factors can be compared across groups/timepoints (Liu et al., 2017). With continuous indicators, this is a sufficient level of invariance if factor means are not being compared (discussion of ordered-categorical indicators below). Third, if metric invariance is established, *strong factorial (i.e., scalar) invariance* can be tested by additionally making item intercepts (i.e., the level of the item, for continuous indicators) or thresholds (i.e., item difficulty, for ordered-categorical indicators, including binary indicators) equivalent over groups. With continuous indicators, scalar invariance implies that it is valid to compare factor means and variances over groups/timepoints (Wu & Estabrook, 2016), as well as factor covariances (across timepoints, since bifactor models have orthogonal factors within a group/timepoint). For example, scalar invariance would be sufficient to interrogate genuine change in mean levels of general psychopathology over development if the model was specified by continuous subscale scores.

With ordered-categorical indicators, many recommend bypassing testing of metric invariance and simultaneously constraining loadings and thresholds, as these jointly influence the probability of an individual choosing a certain category of an item (P.-Y. Chen et al., 2020; L. K. Muthén & Muthén, 2017; Sass et al., 2014; Stark et al., 2006). These simultaneous constraints are consistent with IRT methods to detect DIF, reflect the integral functioning of ordered-categorical items, and have the following advantages. Of foremost concern is that failure to identify non-invariance at the metric level could propagate to errors in subsequent steps (Stark et al., 2006). This is an unnecessary risk to take, since in ordered-categorical data metric invariance does not guarantee invariance of the observed responses (Liu et al., 2017). Furthermore, simulations have shown that the ordered-categorical scalar model is equally sensitive to differences across groups in loadings and thresholds, when compared against an unconstrained configural model (Stark et al., 2006). Finally, fewer comparisons decrease the probability of Type I errors (Stark et al., 2006). However, should researchers wish to specify metric invariance in ordered-categorical data, this requires specification of a marker variable which is loading invariant at all occasions and has at least two invariant thresholds which are not based on sparse data (Liu et al., 2017). An incorrect choice can lead to erroneous conclusions regarding MI, although modification indices can help diagnose this problem. We argue this complexity is unnecessary in light of arguments for jointly constraining loadings and thresholds (P.-Y. Chen et al., 2020; L. K. Muthén & Muthén, 2017; Sass et al., 2014; Stark et al., 2006).

The highest level of MI testing, *residual (or strict) invariance* (also called *unique factor invariance* in ordered-categorical data (Liu et al., 2017)), can be determined by fixing group/timepoint residual (i.e., error) variances to be equal, in addition to equal loadings and means/thresholds. With continuous indicators, if error variances are not equal, groups/timepoints can still be compared on the latent factor, although this is measured with different amounts of error between groups/timepoints (van de Schoot et al., 2012). However, in ordered-categorical data, residual invariance must be met to compare factor means and (co)variances across groups/time (Liu et al., 2017; Millsap & Yun-Tein, 2004). This is because factor models based on ordered-categorical indicators are only indirectly connected to the measured variables—continuous latent responses are inferred from the ordered-categorical indicators based on distributional assumptions. If latent responses are not multivariate normal, invariance in thresholds and loadings will not guarantee MI, and thus changes in the means of the ordered-categorical indicators may not only be attributed to changes in the latent factor (Millsap & Yun-Tein, 2004). There are several reasons for non-invariance and several ways to resolve this. An item may be worded unclearly and thus be inconsistently interpreted. Alternatively, non-invariance may reflect genuine differences over development or across groups. For example, symptoms of restlessness and concentration problems have contributed to scalar non-invariance in longitudinal models of depression in adolescence; at younger ages, these items may be more reflective of difficulties adjusting to school than depressive symptoms (Schlechter et al., 2023). Minor deviations from invariance could be argued to have limited practical consequences on interpretation of the findings (Putnick & Bornstein, 2016). Models with greater non-invariance may imply the latent construct as specified is not comparable across the groups or timepoints in question, and thus the model should be respecified. If configural invariance is not established, exploratory factor analysis, Lagrange multipliers, and Wald tests can be used to identify a properly fitting model across groups/timepoints (Meade et al., 2008). However, it may be possible to establish *partial invariance* of the tested model, where invariance constraints are relaxed for certain parameters (e.g., loadings that vary across groups), thereby controlling for this inequivalence (Vandenberg & Lance, 2000). However, this exploratory process capitalizes on chance and thus should be employed sparingly and with strong theoretical basis. *Bayesian approximate MI* is a promising alternative for models which do not achieve exact invariance. This allows researchers to relax exact equality constraints, and instead assume that parameters are approximately equal, while still maintaining comparability of the underlying constructs (Seddig & Leitgöb, 2018). This approach has been successfully employed in longitudinal CFA (Seddig & Leitgöb, 2018), and in one bifactor model from the review above (Hawes et al., 2018); however, limitations still exist and are discussed below.

### Determining MI in Bifactor Models

We review all the existing literature examining goodness-of-fit indices for discerning MI in bifactor models. As no simulations have been performed in bifactor models with ordered-categorical indicators, the related literature is reviewed. Findings below (summarized in Table 1) are based on simulating multiple levels of invariance to at least the strong level unless specified. The goodness-of-fit indices common to all these studies are comparative fit index (ΔCFI), root mean squared error of approximation (ΔRMSEA), and chi-square difference test (Δχ^2^).

**Table 1.** Summary of Measurement Invariance Cut-Off Findings.

Model, indicator type, invariance type	Simulation findings^a^	Cut-off for (non-) invariance detection	
		ΔCFI	ΔRMSEA
1. Bifactor, continuous, metric (Khojasteh & Lo, 2015)	A. Invariance, *n* = 800–2,400	<.0042 to <0.0033	<.0341 to <.0300
	B. Non-invariance, Δ from λ = 0.7	≥.0033	≥.0300
	- Small (0.2)	- if 8/20 NI on 4/4 specific factors - if 4/20+ NI on ≥ 2/4 specific factors plus general factor	Not reliably detected
	- Large (0.4)	if 4/20+ NI on ≥ 2/4 specific factors	
2. Bifactor, continuous, scalar or residual	**None**		
3. Bifactor, categorical	**None at any level of MI**		
4. First-order, five categories (WLSMV), scalar (Sass et al., 2014)	A. Invariance	<.01	<.01
	B. Non-invariance on 2 or 3/10 items, Δλ or Δτ, or both	≥.002	≥.007
	- Large (0.25)	if n≥600	All scenarios if *n* = 1,000; 5/6 scenarios if *n* = 600
	- Small (0.15)	3/6 scenarios if n≥600	3/6 scenarios if *n* = 1,000
5. First-order, categorical, residual	**None**		
6. First-order, continuous, residual (F. F. Chen, 2007)^b^	A. Invariance, Type I error rate		
	- 0.05	<.005	<.010
	- 0.01	<.01	<.015
	B. Non-invariance: Δλ = 0.4, Δτ = 0.4, Δε = 0.2		
	- *N*≤300, unequal groups	≥.005	≥.010
	- *N* >300, equal groups	≥.01	≥.015

*Note.* CFI = comparative fit index; RMSEA = root mean square error of approximation; NI = non-invariant items; MI = measurement invariance; WLSMV = weighted least squares mean and variance adjusted estimator; (λ = loadings, τ = thresholds/intercepts, ε = residuals.

a Invariance/non-invariance detected with minimal Type I/Type II error. ^b^ΔCFI recommended as main criteria since ΔRMSEA more affected by increasing *n* and model complexity.

#### Studies Using Continuous Indicators

Simulations of first-order models using continuous indicators recommended ΔCFI as the most appropriate goodness-of-fit index for MI (Cheung & Rensvold, 2002). Invariance was not supported when CFI worsened in the constrained model by at least 0.01 (F. F. Chen, 2007; Cheung & Rensvold, 2002), or more strictly, 0.002 (Meade et al., 2008). Invariance cut-offs in bifactor models have only been examined at the metric level. Despite the greater complexity in a bifactor model given cross loadings on the general and specific factors, metric invariance cut-offs for ΔCFI in bifactor models indicated by continuous variables fall within the recommended range for first-order models (0.003–0.004), with slightly less strict values for smaller sample sizes (Khojasteh & Lo, 2015, Table 1). However, to ensure convergence of the bifactor models, the minimum sample size simulated was *n* = 800. Until bifactor simulations on smaller sample sizes are performed, caution is needed in interpreting ΔCFI in bifactor models with small sample sizes. First-order simulations of ΔCFI 0.002 cut-off demonstrated that *n* = 400 would only be sufficiently powered to detect large amounts of non-invariance, *n* = 400 may be reasonable to detect non-invariance if high levels of sensitivity are not required, and power to detect non-invariance was adequate at *n* = 800 (Meade et al., 2008). Such conclusions were also echoed by F. F. Chen (2007), who suggested a stricter ΔCFI cutoff if *n*≤300 (0.005 instead of 0.01). Finally, ΔRMSEA is not recommended for MI testing with continuous indicators both in first-order and bifactor models (Khojasteh & Lo, 2015; Meade et al., 2008).

#### Studies Using Ordered-Categorical Indicators

The above invariance cut-offs for CFI have been found to be acceptable in first-order models indicated by ordered-categorical data, particularly when models are correctly specified, sample sizes are large (≥1,000), and if a small degree of non-invariance is acceptable (Sass et al., 2014). Specifically, when constraining loadings and thresholds simultaneously, F. F. Chen’s (2007) cut-offs adequately identified scalar invariant models (ΔCFI<0.01, ΔRMSEA<0.01). In sample sizes of 1,000, if non-invariance was on at least 20% of the items, Meade et al’s (2008) stricter criteria (ΔCFI≥0.002, ΔRMSEA≥0.007) provided enough power to detect large non-invariance, and some small non-invariance (Sass et al., 2014; Table 1). At smaller sample sizes (*n* = 600), ΔCFI≥0.002 was similarly powered, but ΔRMSEA≥0.007 could not detect all cases where large non-invariance was modeled on 20% of the items. At *n* = 300, both ΔCFI and ΔRMSEA could not detect all cases where large non-invariance was modeled on 30% of the items.

#### Appropriateness of ΔCFI<0.01 MI Cut-Off in Bifactor Models

There are several arguments supporting the more lenient ΔCFI<0.01 invariance cut-offs (F. F. Chen, 2007; Cheung & Rensvold, 2002). The first four arguments apply to using this cut-off in bifactor models with continuous or ordered-categorical indicators, while the last arguments only apply to bifactor models with ordered-categorical indicators. First, models used to devise Meade et al.’s cut-offs have been criticized as being too strict (Little, 2013). Second, cut-offs generated in bifactor models are more liberal than Meade et al.’s cut-offs (Khojasteh & Lo, 2015). Third, large sample sizes (≥1,000) make ΔCFI more prone to rejecting invariance (F. F. Chen, 2007), indicating that a smaller ΔCFI may be overly strict in such instances. Fourth, it is doubtful that a small degree of non-invariance will influence conclusions related to the means of factor scores across groups or time, and so using the above criteria is acceptable (Sass et al., 2014). Fifth, this invariance cut-off appears to adequately identify invariant models in first-order categorical data (Sass et al., 2014). Finally, ΔCFI<0.01 has previously been used to demonstrate invariance in bifactor models with ordered-categorical indicators (Agtarap et al., 2021; Bottesi et al., 2019; Brett et al., 2020; Deutz et al., 2016, 2018; Fong et al., 2022; Forbes et al., 2021; Gluschkoff et al., 2019; Hawes et al., 2018; Porsius et al., 2015), with only one study using Meade’s stricter cut-offs (Grygiel et al., 2019). It is also noted that with ordered-categorical indicators in large sample sizes (*n* = 1,000), ΔRMSEA<0.007 rules out large deviations from non-invariance (Sass et al., 2014). ΔRMSEA should thus be considered alongside ΔCFI but not in smaller samples.

Irrespective of the findings from ΔCFI and ΔRMSEA, we recommend inspecting change in loadings and thresholds over time (or groups). The more complex bifactor structure, consisting of one large general factor with cross-loadings on specific factors, may impede the sensitivity to detect non-invariance (Khojasteh & Lo, 2015). Furthermore, simulations have shown that in single-factor models specified by many ordered-categorical items (*n* = 25), ΔCFI and ΔRMSEA≥0.01 did not always detect scalar non-invariance (D’Urso et al., 2021). This finding is especially relevant for models of psychopathology, which should be specified by a comprehensive set of symptoms from all mental disorders (Lahey et al., 2021).

#### Chi-square-difference test (Δχ^2^)

Δχ^2^ should not be used to demonstrate MI in bifactor models (regardless of indicator type), as this test has the power to detect inconsequential differences between groups in highly complex models (Cheung & Rensvold, 2002), such as bifactor models (Khojasteh & Lo, 2015). This problem is further compounded in models with large sample sizes (Cheung & Rensvold, 2002; Meade et al., 2008). Although the goodness-of-fit indices (e.g., CFI, RMSEA) have also been shown to result in increased rejection of invariance as sample size increases, this was to a lesser degree than for Δχ^2^ (F. F. Chen, 2007). In simulations of MI in bifactor models with continuous indicators, the magnitude of factor loading differences was shown to contribute most to change in goodness-of-fit indices, but sample size contributed most to change in Δχ^2^ (Khojasteh & Lo, 2015). Furthermore, a significant Δχ^2^ does not imply that groups are not comparable, nor does a non-significant finding guarantee the model is not misspecified (Yuan & Chan, 2016). RMSEA<0.05 should be obtained for all increasingly restricted models prior to comparing against a further restricted model, as done in ordered-categorical data (Millsap & Yun-Tein, 2004).

### Residual Invariance

It has been recommended that the residual invariant model be assessed for acceptability of overall fit but not change in goodness of fit statistics (Millsap & Yun-Tein, 2004). Although no goodness of fit cut-offs have been developed for this level of invariance in ordered-categorical data (let alone for higher-order models), ΔCFI<0.01 has been shown to be supportive of residual MI in first-order models with continuous indicators (F. F. Chen, 2007; Cheung & Rensvold, 2002, Table 1). In the absence of more relevant simulations, this cut-off could be cautiously applied to higher-order models with ordered-categorical indicators.

In sum, despite the limited literature on MI in bifactor models, findings from related studies support determining invariance of a such a model based on ΔCFI<0.01. Δχ^2^ is inappropriate for invariance testing of bifactor models due the high complexity of such models, and is even more problematic in large samples. ΔRMSEA is not recommended for MI testing with continuous indicators, but in models specified by ordered-categorical indicators and large sample sizes ΔRMSEA<0.007 is broadly indicative of invariance. When using ordered-categorical indicators, invariance should be assessed to the residual level. Researchers should use the above cut-off guidelines cautiously, acknowledging the multitude of factors that can affect model results (F. F. Chen, 2007; D’Urso et al., 2021; Sass et al., 2014). This degree of skepticism suggests evaluating DIF even if invariance cut-offs are met, something we address in the empirical example. However, we first discuss estimator choice and influence of missing data in MI testing.

### Estimator Choices and Missingness in MI Testing

With continuous data, full information maximum likelihood (ML) estimation is favored for CFA, as it efficiently produces unbiased parameter estimates in normally distributed data, and robust methods (MLR) can address deviations to normality (Zhong & Yuan, 2011). Of the estimators appropriate for ordered-categorical data, none are perfectly suited to MI testing (Table 2). However, the weighted least squares mean and variance adjusted estimator (WLSMV) appears most appropriate for MI testing with polytomous data, where the testing of residual invariance is necessary (Liu et al., 2017; Millsap & Yun-Tein, 2004). Residual MI testing is not currently possible with categorical ML estimation due to the computationally intensive numerical integration required (B. O. Muthén et al., 2015). Conversely, Bayes has not yet been developed for threshold invariance testing in polytomous data (B. O. Muthén et al., 2015). In contrast to other estimators, WLSMV’s computational burden does not increase exponentially with an increase in factors or sample size, but models specified by many items may be uniquely laborious for WLSMV (B. O. Muthén et al., 2015). Thus, single-group longitudinal MI testing may be particularly problematic for WLSMV, as the number of items is multiplied by the number of waves tested. This highlights the need for Bayesian and ML estimators to be developed to test MI using polytomous data, allowing researchers to choose an estimator which minimizes computational burden for a given model.

**Table 2.** Properties of Estimators Appropriate for Ordered-Categorical Data^a^.

Statistical package^b^	WLSMV	ML-integration^c^	ML-MHRM	Bayesian
	Mplus, lavaan	Mplus, MIRT	MIRT	Mplus, Blavaan
Properties relevant to MI testing^b^				
Ability to model threshold invariance in polytomous data	Yes	Yes	Yes	**No, binary only**
Approximate fit statistics for comparing nested models	Yes	Yes	Yes	**Possibly** ^d^
Ability to model residual variances and covariances	Yes	**No**	**No**	Yes
Other relevant properties				
Full-information estimator	**No**	Yes	Yes	Yes
Polytomous data estimation speed equivalent to binary	Yes	**No—slower**	Yes	**No—slower**
Binary empty cells may be problematic	**Yes**—see Supplement 1	No	No	No
Computational burden				
Increases exponentially with a linear increase in factors (4+ problematic)	No	**Yes**	No	No
Increases exponentially with a linear increase in variables (50+ problematic)	**Yes**	No	No	No
Increases in large samples (e.g. 1,000+)	No	**Yes**	**Yes**	**Yes**

*Note.* WLSMV = weighted least squares mean and variance adjusted; ML = maximum likelihood; MHRM = Metropolis-Hastings Robbins-Monro algorithm; MI = measurement invariance; CFI = comparative fit index; RMSEA = root mean square error of approximation.

a Unless specified, findings for MHRM from Cai (2010), other estimator findings from B. O. Muthén et al. (2015). ^b^ We focus on two widely-used packages: Mplus (https://www.statmodel.com) and R with packages lavaan (https://cran.r-project.org/web/packages/lavaan/lavaan.pdf), MIRT (Multidimensional Item Response Theory, https://cran.r-project.org/web/packages/mirt/mirt.pdf), and Blavaan (https://cran.r-project.org/web/packages/blavaan/blavaan.pdf). ^c^ with numerical integration using fixed or adaptive quadrature (Mplus and MIRT for details). In Mplus, ML-categorical is always robustly estimated to account for non-normality. ^d^ Bayesian analogues for approximate fit statistics (e.g., CFI, RMSEA) are comparable to ML values, so ML guidelines for overall model fit apply, but MI cut-off guidelines may not (Garnier-Villarreal & Jorgensen, 2020). Posterior predictive p-value (PPPV) is analogous to chi-square, so comparable Type I errors in large sample sizes.

Of the estimators reviewed, WLSMV is the only limited information estimator, so parameter estimates and tests of model fit could be biased if data are missing at random (Liu et al., 2017). However, with sample sizes of 500 or 1,000, and missing data rates up to 50% across groups, scalar MI simulations showed that WLSMV resulted in acceptably small levels of mean relative bias in loading estimates and their standard errors across varying levels of invariance in thresholds and loadings (P.-Y. Chen et al., 2020). When sample sizes were small (*n* = 300), standard errors were biased only when missingness was 50%, but were acceptable at 30% missingness. Multiple imputation in data of various distributions can mitigate missingness bias, yet currently this cannot resolve MI testing issues in large samples and complex models. Thus far, only approaches to pooling Δχ^2^ from multiply imputed datasets have been tested (Liu & Sriutaisuk, 2021). Finally, predictors of missingness can be included in WLSMV models to help mimize missingness bias (Asparouhov & Múthen, 2010), yet this can introduce infeasible levels of computational burden in models with many indicators (B. O. Muthén et al., 2015).

Even with ML and continuous data, simulations have shown that severely unbalanced groups (e.g., due to missing data over time) can mask non-invariance (Yoon & Lai, 2018). However, when testing scalar invariance when one group was half the size of the other, ΔRMSEA was not adversely affected. Under these conditions, scalar non-invariance was found to improve ΔCFI to a small degree (0.005), but this was only tested in small sample sizes (groups of 200 and 400 (Yoon & Lai, 2018). These findings underscore the importance of assessing several fit indices when drawing conclusions regarding invariance between unbalanced groups. Although prior simulations did not recommend ΔRMSEA for MI testing with continuous indicators, only equal sample sizes were tested (Khojasteh & Lo, 2015; Meade et al., 2008). With unbalanced groups, this fit index appears to be optimal.

To summarize, WLSMV is recommended for MI testing using polytomous data, being the only estimator that currently allows residual invariance testing in such variables. However, the computational burden for WLSMV makes estimation slow and possibly infeasible for single-group longitudinal MI testing of large models. WLSMV has been shown to be acceptable for scalar MI testing in ordered-categorical data with missing data rates up to 50% in sample sizes of 500 or more. Even with ML and continuous data, researchers testing MI should be wary that a large degree of size imbalance across groups may affect ΔCFI, although ΔRMSEA does not appear to be affected.

Thus far we have reviewed the importance of establishing longitudinal MI in bifactor models, provided guidance on MI cut-offs to employ when ordered-categorical indicators are utilized, and outlined estimator choices and missing data considerations. We now focus on bifactor models of psychopathology over two or more waves of data as a case study for how adequately this literature has addressed MI to date.

### Limitations of MI Testing in Bifactor Models of Psychopathology

We reviewed confirmatory bifactor models of psychopathology (the “*p*” factor, or simply “*p*”) encompassing internalizing and externalizing domains, the most-studied key underlying processes in psychopathology (Caspi & Moffitt, 2018). Studies reporting bifactor models generated in at least two waves were assessed for demonstration of longitudinal MI. To date, few existing bifactor models of psychopathology have established strong longitudinal MI. Some studies assessed stability or correlation of factors over time based on different models at each wave, without having established even the lowest level of MI (Class et al., 2019; Deutz et al., 2020; Greene & Eaton, 2017). More studies demonstrated longitudinal configural invariance when the same confirmatory model yielded acceptable fit at each wave (Castellanos-Ryan et al., 2016; Deutz et al., 2016, 2018; Forbes et al., 2021; Gluschkoff et al., 2019; McElroy et al., 2018; Noordhof et al., 2015; Olino et al., 2018; Snyder et al., 2017). However, many studies did not impose further MI tests (McElroy et al., 2018) or adequately test for or demonstrate strong MI (Castellanos-Ryan et al., 2016; Noordhof et al., 2015; Olino et al., 2018; Snyder et al., 2017). In studies where strong MI has not been demonstrated over time, the meaning of the construct and levels of the underlying items are not known to be equal across time points (van de Schoot et al., 2012). Therefore, interpretation of the longitudinal associations in such models is questionable.

Inconsistent criteria have been applied in the few studies of *p* which have tested longitudinal MI beyond configural invariance. One tested for strong invariance by constraining factor loadings and thresholds to be equal over three waves (Noordhof et al., 2015). Although this model exhibited good fit, it was not compared with an unconstrained model, and thus invariance is unknown. Three studies modeling continuous indicators tested for metric invariance (weak factorial invariance) by constraining the factor loadings to be equal over time (Castellanos-Ryan et al., 2016; Olino et al., 2018; Snyder et al., 2017). Snyder et al’s model was supportive of metric invariance, but they did not then test strong invariance. The remaining studies rejected metric invariance, but for different reasons: the metric model yielded unacceptable fit (Olino et al., 2018), or Δχ^2^ indicated that constrained models had significantly worse fit than unconstrained models (Castellanos-Ryan et al., 2016). Although lack of metric invariance is undeniable in Olino and colleagues’ study, the latter study had a large sample size (>2,000): under such circumstances, Δχ^2^ is highly sensitive to inconsequential differences, and thus Δχ^2^ may not be an accurate indicator of invariance (F. F. Chen, 2007; Cheung & Rensvold, 2002; Meade et al., 2008). When a more widely accepted indicator of invariance was utilized (change in Comparative Fit Index, ΔCFI ≤0.01 (Putnick & Bornstein, 2016), strong longitudinal MI was established in bifactor models specified by ordered-categorical variables (Deutz et al., 2016, 2018; Forbes et al., 2021; Gluschkoff et al., 2019).

When testing MI, none of the above studies discussed how the dual factor loadings in the bifactor model may influence MI cut-offs. Of the models tested beyond configural MI specified by ordered-categorical indicators, only half mentioned the limitations of current MI cut-offs for this type of data. None tested residual invariance, as has been argued is required in ordered-categorical data (Liu et al., 2017; Millsap & Yun-Tein, 2004). This exemplifies the importance of the present review of MI cut-offs in bifactor models to provide guidelines for applied researchers who utilize such models in future. We now turn to an empirical example to illustrate how these guidelines can be employed.

## Empirical Example

**Figure 2.**
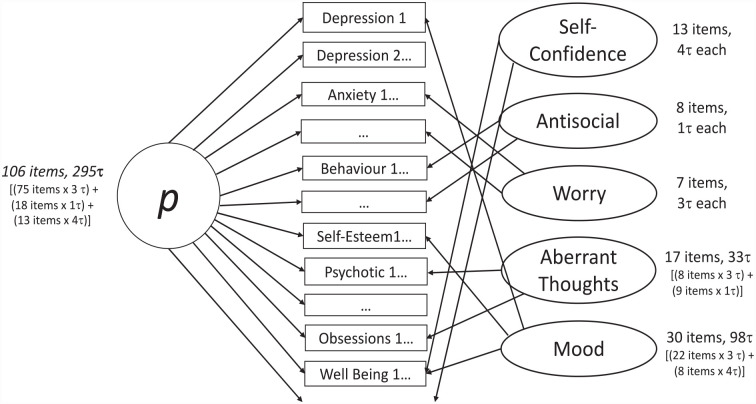
Bifactor Model From Empirical Example. *Note. p =* general factor of psychopathology; τ = thresholds (item categories minus 1). Specific factors were named based on item loadings (St Clair et al., 2017; Supplemental Table S1). Items are illustrative of the 106 items in the model; the positive methods specific factor is omitted for simplicity.

The present example longitudinally extends a model published on baseline data of 106 items from measures of depressive, anxiety, obsessive, antisocial behavioral, and psychotic-like symptoms, as well as self-esteem and well-being (St Clair et al., 2017). At baseline, compared with first-order (single-factor and correlated factor) models and a second order model, the most theoretically plausible (and best-fitting) model was a Schmid-Leiman bifactor transformation (Brown, 2006) of a five-factor Confirmatory Factor Analytic model (i.e., a general factor, *p*, with five orthogonal specific factors; Figure 2; St Clair et al., 2017). This model was more theoretically plausible than other models for reasons such as: the correlated factor models had unacceptably high correlations (*r* > .85) between the factors, indicating a general factor underlying all factors; the model with four specific factors had antisocial items loading with obsessions, compulsions, and psychotic symptoms—and there is no precedence for antisocial symptoms loading with the latter symptoms. All factors of the final model were set to be orthogonal (uncorrelated), as shared variance is captured in the general factor (Lahey et al., 2021). In addition, a positive method specific factor was included to account for items’ positive/negative wording. This better addresses the different framing of questions than simply recoding positively worded items as others have done (McElroy et al., 2018) and facilitates accurate interpretation of factors (Gignac, 2007).

We theorized that the present bifactor model would demonstrate strong MI, as found in prior bifactor models of psychopathology which yielded specific internalizing and externalizing factors (gender invariance (Deutz et al., 2016, 2018)) and longitudinal invariance (Deutz et al., 2016, 2018; Forbes et al., 2021; Gluschkoff et al., 2019).

### Participants

About 2,403 adolescents and young adults aged 14–24 (54% female, *n* = 1,287) were recruited in the United Kingdom from Cambridgeshire and London, comprising the NeuroScience in Psychiatry Network (NSPN) cohort (Kiddle et al., 2018). This sample was broadly representative on socio-demographic features of this age group from English and Wales census data (Kiddle et al., 2018). Ethical approval was obtained from the National Health Service Research Ethics Service (#97546). The self-report items in the bifactor model were obtained from a home questionnaire pack mailed to participants’ home. Three waves were collected annually (on average, Wave 2 was collected 13.5 months (*SD* = 3.64) after Wave 1, and Wave 3 was collected 27.2 months (*SD* = 3.20) after Wave 1).

### Measures

The provenance of items used in the bifactor model has been described previously (St Clair et al., 2017); Supplemental Table S1 provides a summary. Items were ordinal or binary and thus were specified as ordered-categorical in analyses. Study data were collected and managed using REDCap electronic data capture tools hosted at the University of Cambridge, UK (Harris et al., 2009, 2019). REDCap (Research Electronic Data Capture) is a secure, web-based software platform designed to support data capture for research studies, providing (a) an intuitive interface for validated data capture; (b) audit trails for tracking data manipulation and export procedures; (c) automated export procedures for seamless data downloads to common statistical packages; and (d) procedures for data integration and interoperability with external sources. Data and additional information on measures can be requested here: https://nspn.org.uk.

### Statistical Analyses

#### Bifactor Modeling

The Schmid-Leiman transformation was performed as outlined above. For model identification, factor variances were fixed to one, and factor means fixed to zero (Liu et al., 2017; L. K. Muthén & Muthén, 2017). Another approach to model identification is to fix a factor loading to one, and constrain the intercept/threshold for that same variable to zero (Liu et al., 2017). However, if during MI testing a non-invariant parameter is constrained, this can result in model misfit and erroneous conclusions regarding MI (Liu et al., 2017). To avoid such problems, the former approach was chosen.

#### Invariance Testing

To test *gender invariance*, the optimal Schmid-Leiman transformation reported at baseline (St Clair et al., 2017) was performed separately in each gender at baseline to test appropriateness of fit in each group (Meade et al., 2008). Consistent with the original modeling, theoretically relevant modifications which were applicable to both genders were considered for low loadings (<0.30 for the general factor and <0.15 for specific factors) and high modification indices (>100, due to the large number of participants in the present sample (St Clair et al., 2017). A theoretically relevant modification could, for example, be to drop a low loading item from a specific factor if the item was not unambiguously conceptually related to the other items in that specific factor.). Modifications meeting these criteria were applied to all subsequent models. Gender invariance was tested using a multigroup confirmatory factor analytic framework (van de Schoot et al., 2012). Both genders were modeled together following the increasingly restrictive invariance tests outlined below.

For establishing *longitudinal invariance*, the model was first performed separately at each wave to test for appropriateness of fit over time (Meade et al., 2008). Following this, a single-group model (wide formatted data) with 318 items from Waves 1–3 was tested. As this 21-factor model did not converge, single-group MI was tested in a piecewise fashion with two waves at a time. These models were contrasted against a multigroup model, as all waves were able to be tested at once in long formatted data. We then compared computational burden and MI findings from the piecewise single-group models versus the one multigroup model. Although the single-group CFA better accounts for the dependent nature of the data, the much larger array of data can result in improper solutions, particularly for models with many items (Vandenberg & Lance, 2000), as we observed. Furthermore, multigroup simulation findings indicate that, at the level of imbalance, we observe in groups due to attrition (up to 50%), estimates and standard errors are not biased, and ΔRMSEA and most likely ΔCFI are appropriate tests of MI (P.-Y. Chen et al., 2020; Yoon & Lai, 2018). To our knowledge, no one has contrasted longitudinal MI findings from single-group versus multigroup models. However, given the importance in structural psychopathology research of specifying larger models with more comprehensive sets of symptoms modeled over the lifespan (Caspi & Moffitt, 2018; Lahey et al., 2021), we anticipate others will also be faced with convergence issues when testing longitudinal MI in single-group models. Thus, the possibility of equivalent identification of longitudinal MI in a multigroup model is important to explore. Finally, metric invariance was not tested on its own given the multiple advantages to constraining loadings and thresholds simultaneously, as we previously reviewed. Adhering to the key study elucidating MI cut-offs in ordered-categorical data (Sass et al., 2014), we simultaneously constrained these parameters and compared fit statistics against the configural model. This allows contextualizing the present findings with these relevant simulations.

*Specification* of the increasingly constrained models was as follows. All models used the WLSMV estimator with theta parameterization and a probit link, as appropriate for invariance testing of ordered-categorical indicators (Millsap & Yun-Tein, 2004). For the configural model, thresholds and factor loadings were free across groups, factor variances and residual variances were fixed at one in all groups, and factor means fixed at zero in all groups (L. K. Muthén & Muthén, 2017). Correlated residuals were modeled identically at each wave (Joo & Kim, 2019) for indicators that were related but distinct (St Clair et al., 2017). For scalar and residual invariance models, the factor variances were fixed to one in one group and freed in the other group(s), and factor means were fixed to zero in one group and freed in the other group(s) (L. K. Muthén & Muthén, 2017). Both models had factor loadings and thresholds constrained to be equal across groups. In the scalar model, residual variances were fixed to one in one group and freed in the other group(s), but the residual invariance model had residual variances fixed to one in all groups. Residuals were correlated over time for modification indices >100.

*Criteria to assess measurement invariance*: To assess invariance, a CFI difference between the scalar and configural model of <0.01 was required (F. F. Chen, 2007; Cheung & Rensvold, 2002), as justified in the preceding review. Given the large sample size and ordered-categorical data, we also considered ΔRMSEA<0.007 supportive of invariance (Sass et al., 2014). Correct specification of each increasingly restrictive model was ensured by requiring RMSEA<0.05 for all models (Millsap & Yun-Tein, 2004; Yuan & Chan, 2016). As no goodness of fit cut-offs has been developed for residual invariance in ordered-categorical data (let alone for higher-order models), the residual invariant model was primarily assessed for acceptability of overall fit (Millsap & Yun-Tein, 2004), with a ΔCFI<0.01 compared to the scalar model additionally supportive of residual invariance (F. F. Chen, 2007; Cheung & Rensvold, 2002).

To address concerns that ΔCFI and ΔRMSEA may not adequately detect parameter differences across groups in factors specified by many items (D’Urso et al., 2021), the magnitude of loading and threshold differences over time were assessed regardless of whether these fit indices supported invariance. To quantify non-equivalence, we considered the following. First, we calculated the average difference when invariance violations were at least small (loading differences 0.10+ and threshold differences 0.25+, D’Urso et al., 2021; Nye et al., 2019). These are smaller violations than many have used to measure non-equivalence in models which are not bifactor (P.-Y. Chen et al., 2020; Guenole & Brown, 2014; Liu & Sriutaisuk, 2021; Sass et al., 2014; Stark et al., 2006). However, bifactor models typically have smaller loadings due to cross-loadings on the general and specific factors (e.g., all models of *p* reviewed herein have loadings ≤0.2 except Caspi et al., 2014). Thus, in the bifactor model, smaller loading differences across time or groups are likely to reflect a greater deviation from the original loading and thus be more likely to be indicative of non-invariance. Second, as non-equivalence could be mixed (occur in opposite directions, e.g., subsequent wave could have lower or higher loadings) the average difference based on absolute values was calculated to clarify when non-equivalence was negligible versus canceled out (Nye et al., 2019). Finally, we note when loading differences are 50% or more of the earlier wave’s loading, as this level of non-invariance may introduce unacceptable levels of bias (Guenole & Brown, 2014).

All analyses were performed in Mplus Version 8 (L. K. Muthén & Muthén, 2017). Code is available here: osf.io/fbd3h.

### Results

#### Missing Data

As for the baseline model (St Clair et al., 2017), participants’ data were included in the bifactor model at each wave if they completed 85% of the original 118 items, and 85% of each original measure (Supplemental Table S1). Of the 2,403 participants, 99% (*n* = 2,372), 69% (*n* = 1,659) and 46% (*n* = 1,096) completed sufficient data to compute the bifactor model at waves 1–3 respectively. All available data were used for MI testing, resulting in unequal sample sizes between gender groups and over time (14% fewer males than females [1,099 and 1,273 respectively]; 30% missing at Wave 2 vs Wave 1; 54% missing at Wave 3 vs Wave 1). Simulations have shown that this level of imbalance between groups is not highly problematic for the ΔCFI or ΔRMSEA used in invariance testing (Yoon & Lai, 2018). At this level of missingness, WLSMV yields acceptably small levels of mean relative bias in loading estimates and their standard errors (P.-Y. Chen et al., 2020). Therefore, it was appropriate to estimate all models with WLSMV using raw data. As noted in “MI Testing in Bifactor Models”, multiple imputation or adding predictors of missingness in WLSMV are not yet feasible options for MI testing in large, complex models (Asparouhov & Múthen, 2010; Liu & Sriutaisuk, 2021; B. O. Muthén et al., 2015).

#### Measurement Invariance

Following running the original model on the full baseline dataset (Supplement 1), the configural gender multigroup model was tested. This failed to converge on baseline data, so each gender was modeled separately to identify sources of misfit. All loadings were above cut-offs in the male model, but the female model had a low loading (0.126) of MFQ24 (“I was a bad person”) on the antisocial behavior specific factor. Given the ambiguous wording of this item (e.g., endorsement could reflect low self-esteem instead of antisocial behavior), it seemed theoretically appropriate to drop this loading on the specific (but not the general) factor. Upon doing so, the configural multigroup model for gender converged. (Only one other loading in females was minimally below cut-off [0.24 on general factor], and thus was retained.) This modified model yielded excellent fit in separate models for each gender (Table 3), with no additional loadings below cut-offs, and no items having modification indices above our cut-off of 100. The model also yielded excellent fit in the whole sample at each timepoint (Table 3), with all loadings significant and above cut-offs (Supplemental Table S2). There were no item loadings with modification indices>100 at all three timepoints. Sparse data in one Wave 3 item had minimal impact on the model (Supplement 1).

**Table 3.** Fit Indices for Measurement Invariance Testing of the Bifactor Model of Psychopathology (*p*) from the Empirical Example.

Model	*N*	Chi square (χ^2^)	*Df*	Parameters	CFI	TLI	RMSEA
Baseline only models							
Full sample	2,372	16,785	5,351	509	0.955	0.953	.030
Females only	1,273	10,935	5,351	509	0.960	0.959	.029
Males only	1,099	8,802	5,351	509	0.968	0.967	.024
Gender invariance	2,372						
Configural		19,594	10,717	1,003	0.964	0.963	.026
Scalar		20,293	11,083	637	0.963	0.963	.026
Residual		18,149	11,204	516	0.972	0.972	.023
Wave 2 model	1,659	12,062	5,351	509	0.970	0.969	.027
Wave 3 model	1,096	9,229	5,351	509	0.974	0.973	.026
Waves 1 and 3^a^ invariance single-group	2,382						
Configural		30,373	21,933	1,023	0.975	0.974	.013
Scalar		30,599	22,307	649	0.975	0.975	.012
Residual		30,144	22,413	543	0.977	0.977	.012

*Note.* CFI = comparative fit index; TLI = Tucker–Lewis index; RMSEA = root mean square error of approximation.

a Other wave comparisons in Supplemental Table S3. Only homotypic paths were modeled; fit indices where heterotypic paths were included are shown in Table S3.

Model fit was excellent for configural, scalar, and residual invariance models across gender and time, using both the single-group and multigroup approaches for time (Table 3, Supplemental Table S3). In the single-group model, only two obsessive-compulsive items required residual correlations across waves. Across gender and time, comparing scalar and residual invariance models with the next least constrained model (configural and scalar, respectively), changes in CFI and RMSEA were less than even the strictest criteria (Meade et al., 2008): CFI declined at most by .001 and at worst RMSEA stayed the same (Table 3, Supplemental Table S3). Thus, residual MI was supported using both the single-group and multigroup approach. However, testing the final single-group models resulted in 43 days more computation time than the multigroup approach (Supplement 1).

In the single-group model, the above findings are based on models where factor autocorrelations were allowed while cross-factors were modeled orthogonally over time. This is because when heterotypic paths were allowed, models yielded equivalent fit, and also supported residual invariance (Supplemental Table S3). Therefore, the more parsimonious model was preferred, in line with prior research (Gluschkoff et al., 2019). However, significant but small (.08–.26, *p*<.01) cross-lagged standardized effects were observed from Wave 1 to 3 with residual invariance modeled (Supplemental Table S4). Findings were generally comparable when scalar invariance was modeled; however, one significant cross-lagged effect in the scalar model became non-significant in the residual model. Reciprocal effects were observed across *p* and aberrant thoughts specific factor, and antisocial and worry specific factors (negative effect). Unidirectional effects were observed from aberrant thoughts to antisocial specific factor and mood to self-confidence specific factor.

Assessing change in parameters over time revealed that most factors over all waves exhibited an average of small non-invariance or less in under a quarter of items (Table 4). Mixed non-invariance in *p*, aberrant thoughts and mood specific factors contributed to some of the negligible non-invariance observed. The aberrant thoughts and antisocial specific factors exhibited an average of small threshold differences from Waves 1 to 3 in approximately 50% and 75% of their respective thresholds. The antisocial specific factor also had small or more loading differences in at least half of the items for Waves 1–3 and 2–3 comparisons. However, only three loading differences (each for a different factor) were approximately half the size of the earlier wave’s loading, and these items had negligible threshold differences.

**Table 4.** Summary of Non-Invariant^a^ Parameters Over Time from the Empirical Example Bifactor Model of Psychopathology (*p*).

Factor	Loading differences (Δλ)			Threshold differences (Δτ)		
	w2—w1	w3—w2	w3—w1	w2—w1	w3—w2	w3—w1
*p* (general factor)						
Parameters/total	4/106	5/106	9/106	8/295	9/295	68/295
Range (ABS)	0.103–0.171^b^	0.106–0.276	0.096–0.270	0.255–.327	0.246–0.424	0.246–0.499
ABS average	0.126	0.153	0.124	0.281	0.298	0.311
Average^c^	0.126	–0.043	–0.025	0.200	0.298	0.247
% non-invariant	3.8%	4.7%	8.5%	2.7%	3.1%	23.1%
Summary	<5% small	Negligible mixed	Negligible mixed	Negligible mixed	<5% small	<25% small
sf1 (self-confidence)						
Parameters/total	0	0	1 /13	0	0	4/52
Range (ABS)			0.106			0.249–0.379
ABS average			0.106			0.286
Average			–0.106			–0.286
% of total factor			7.7%			7.7%
Summary	Negligible	Negligible	<1/12 small	Negligible	Negligible	<10% small
sf2 (antisocial)						
Parameters/total	0	4/8	5 /8	1/8	1/8	6/8
Range (ABS)		0.155–0.227^b^	0.096–0.223	0.259	0.274	0.275–0.481
ABS average		0.188	0.142	0.259	0.274	0.362
Average		0.188	–0.142	0.259	0.274	0.362
% of total factor		50%	62.5%	12.5%	12.5%	75%
Summary	Negligible	50% medium	< 2/3 small	1/8 small	1/8 small	75% small-med
sf3 (worry)						
Parameters/total	0	1/7	0	0	0	1/21
Range (ABS)		0.098				0.302
ABS average		0.098				0.302
Average		0.098				0.302
% of total factor		14.3%				4.8%
Summary	Negligible	1/7 small	Negligible	Negligible	Negligible	<5% small
sf4 (aberrant thoughts)						
Parameters/total	3/17	2/17	3/17	2/33	3/33	15/33
Range (ABS)	0.090–0.131	0.102–0.115	0.108–0.220	0.260–0.317	0.264–0.304	0.246–0.419
ABS average	0.121	0.109	0.146	0.289	0.284	0.313
Average^c^	–0.033	0.109	–0.073	0.289	0.284	0.313
% of total factor	17.7%	11.8%	17.7%	6.1%	9.1%	45.5%
Summary	Negligible mixed	< 1/8 small	Negligible mixed	<1/12 small	<10% small	<50% small
sf5 (mood)						
Parameters/total	1/30	1/30	1/30	0	0	11/98
Range (ABS)	0.121	0.102	0.147^b^			0.248–0.379
ABS average	0.121	0.102	0.147			0.275
Average^c^	–0.121	0.102	0.147			–0.073
% of total factor	3.3%	3.3%	3.3%			10.2%
Summary	<5% small	<5% small	<5% small	Negligible	Negligible	Negligible mixed

*Note.* sf = specific factor (positive loading sf not discussed as this is purely a methods factor); ABS = absolute value.

a Non-invariance: Δλ 0.10 = small, 0.20 = medium, 0.30 = large; Δτ 0.25 = small, 0.50 = medium, 0.75 = large (Nye et al., 2019). ^b^ highest Δλ (1 item) is ~50% of earlier wave loading (45%+ flagged, range=47%-52%) but Δτ is < small (≤0.232). This level of loading invariance is very unlikely to substantially affect findings in structural regression models (Guenole & Brown, 2014). ^c^ Averages may be below non-invariance cut-offs due to mixed differences in parameters (some positive, some negative).

### Discussion

This empirical example extends the limited prior work on gender and longitudinal invariance of bifactor models. Of the existing studies of bifactor models of psychopathology, only a small proportion have demonstrated strong invariance (gender invariance (Deutz et al., 2016) and longitudinal invariance (Deutz et al., 2016, 2018; Forbes et al., 2021; Gluschkoff et al., 2019)), all of which utilized ordered-categorical indicators. These prior studies were conducted throughout childhood and early adolescence (Deutz et al., 2016, 2018) and adulthood (Forbes et al., 2021; Gluschkoff et al., 2019), whereas the present study covers the period from adolescence into young adulthood, when mental illness steeply increase (Kessler et al., 2007). In addition to fitting the data well at each of the three timepoints, the present model appears to be equivalent across males and females, and over 3 years of measurement. The empirical example reveals the first model of *p* to establish longitudinal residual invariance, using the guidelines advocated above. This higher level of invariance is required for demonstrating MI in factor models with ordered-categorical items, indicating that changes in the items over time are attributable to changes in the latent factors over time (Liu et al., 2017; Millsap & Yun-Tein, 2004). This strengthens the validity of any longitudinal associations to be made more than prior longitudinal studies of *p*. The same applies to any conclusions to be made about gender differences, given the demonstrated residual gender invariance of the empirical example.

Although ΔCFI and ΔRMSEA supported residual longitudinal MI, inspecting change in loadings and thresholds revealed a more nuanced picture. Across all waves, most factors exhibited negligible levels of non-invariance, supporting their comparability over time (Nye et al., 2019). The aberrant thoughts and antisocial specific factors exhibited threshold differences which could account for discernable effects (Nye et al., 2019). However, this conclusion is tentatively based on prior simulations of five-category indicators with no cross-loadings (Nye et al., 2019); interestingly, the factors with discernable threshold differences were the only ones specified by a majority of binary items. This level of non-invariance may in fact have limited practical consequence: much larger threshold non-invariance was simulated to detect group bias in structural regression models, and all observed loading differences were well below discernable levels (Guenole & Brown, 2014). Other bifactor models of *p* which have established strong MI by ΔCFI<0.01 are not immune to item-level non-invariance (Deutz et al., 2018; Forbes et al., 2021; Gluschkoff et al., 2019). For example, 60% of a factor’s items showed large non-invariance (0.3+; Deutz et al., 2018). In all cases, as in the present study, the largest non-invariance was seen in specific factors. This points to the importance of careful inspection of parameter changes over time (or groups) in bifactor models even if MI cut-offs have been met. Simulations are then needed to guide researchers on the practical impact of various levels of non-equivalence in bifactor models (Nye et al., 2019). Simulations also need to model the impact of item heterogeneity in threshold non-invariance, evident in our empirical example (e.g., only one of three thresholds for an item was non-invariant). With one exception (Guenole & Brown, 2014), the above simulations all modeled non-invariance as a uniform shift across all thresholds in an item (P.-Y. Chen et al., 2020; D’Urso et al., 2021; Liu & Sriutaisuk, 2021; Nye et al., 2019; Sass et al., 2014; Stark et al., 2006).

The empirical example highlights the computational demands of single-group MI testing of large, complex models with multiple waves. Here, simultaneously testing three waves in a single-group model (of 318 items) resulted in non-convergence, a potential consequence of a large data array (Vandenberg & Lance, 2000). The bifactor models of *p* reviewed above which tested single-group MI only had two waves (Castellanos-Ryan et al., 2016; Deutz et al., 2016; Forbes et al., 2021; Gluschkoff et al., 2019; Olino et al., 2018), which we also found feasible. Therefore, assessing the present model using a single-group framework required piecewise testing of three two-wave models. This approach was very computationally costly, resulting in 43 more computation days compared with the multigroup approach where all three waves were tested simultaneously. Although the single-group approach is favored as it accounts for the longitudinal relationships between repeated measurements (Vandenberg & Lance, 2000), in this example both approaches yielded the same MI conclusion. We are not aware of any simulation studies which have explored under which conditions this conclusion would hold. The present findings argue for such simulations to be performed, to clarify whether a multigroup model is indeed an appropriate approach for longitudinal MI testing in large and complex models where a single-group model is infeasible. Finally, as the empirical example was performed using WLSMV, the findings illustrate the need for development of the Bayesian estimator for MI testing of polytomous items. Bayes has lower computational demands for a large number of variables than WLSMV (B. O. Muthén et al., 2015), and in multidimensional models of binary data, has shown the greatest convergence rates and lowest parameter bias, followed by WLSMV, and then ML (Garnier-Villarreal et al., 2021).

Although the single-group model allows testing of heterotypic paths, many studies from our review of MI testing in bifactor models did not assess this (Brett et al., 2020; Deutz et al., 2018; Fong et al., 2022; Forbes et al., 2021; Grygiel et al., 2019; Hawes et al., 2018; Porsius et al., 2015). Most which did include heterotypic paths did not report the magnitude or significance of these associations (Agtarap et al., 2021; Bottesi et al., 2019; Deutz et al., 2016). Only the present study and one other compared models allowing only homotypic paths versus those allowing heterotypic paths: in both cases fit was equivalent, and MI conclusions were the same from either model, and thus the more parsimonious model was preferred (Gluschkoff et al., 2019). When testing longitudinal MI in single-group bifactor models, we recommend heterotypic paths be assessed and justification be provided for which model is ultimately pursued. Ideally, heterotypic estimates should be reported as part of MI testing, as these may be insightful for subsequent research, for example, to help understand continuity and change in psychopathology over the lifespan.

Bifactor models which do report heterotypic paths should demonstrate longitudinal MI to increase confidence that estimates are reflective of true heterotypy instead of artifact from non-invariance. This is particularly important as significant heterotypic paths are small (standardized estimates from 0.06 to 0.26 in the present and prior studies) (Castellanos-Ryan et al., 2016; Deutz et al., 2020; Gluschkoff et al., 2019; McElroy et al., 2018). To our knowledge, these path coefficients have only been reported in bifactor models of psychopathology. Ideally, the same level of MI should be used for comparability across the literature. Differing levels of MI being modeled may change conclusions regarding heterotypic paths, as we observed. Of the prior studies of *p* which have reported heterotypic paths, only one exhibited scalar invariance (Gluschkoff et al., 2019), and metric invariance (Snyder et al., 2017), three demonstrated configural invariance (Castellanos-Ryan et al., 2016; McElroy et al., 2018; Olino et al., 2018), and three did not even meet that threshold, using different models at each wave (Class et al., 2019; Deutz et al., 2020; Greene & Eaton, 2017). Thus, only the first two studies, indicated by ordered-categorical and continuous variables respectively, exhibited appropriate levels of MI to compare factor covariances over time. Study comparability seems further hampered by power: significant heterotypic paths have been observed in large samples (*n*>1,000) including the present study (Castellanos-Ryan et al., 2016; Deutz et al., 2020; Gluschkoff et al., 2019; McElroy et al., 2018), but not in smaller samples (*n*<600), although standardized estimates (>.1) were comparable or larger to some which were significant in the bigger studies (Class et al., 2019; Olino et al., 2018; Snyder et al., 2017). Further adding to this heterogeneity, bifactor models of *p* can cover different symptom domains, and thus the interpretation of *p* can vary across studies, with different specific factors observed, under varying degrees of reliability (Watts et al., 2020). Consistent testing of longitudinal MI is necessary to address one source of heterogeneity in this literature, before firmer conclusions can be made regarding heterotypic change in *p* and specific factors over development.

## General Discussion

This article highlights key issues in assessing bifactor models with respect to MI. We underscore the importance of MI testing in bifactor models and current gaps which hinder such testing. Our literature review revealed that few studies have assessed longitudinal MI in bifactor models. The paucity of guidelines for how to determine MI in bifactor models resulted in inconsistency in what level MI was tested to, and which fit indices and cut-offs were used for determining MI. This is exemplified in the literature on the *p*-factor, where strong longitudinal MI testing was omitted or inappropriately applied in longitudinal studies of *p* (Castellanos-Ryan et al., 2016; McElroy et al., 2018; Noordhof et al., 2015; Snyder et al., 2017). This results in questionable interpretations of latent means and factor correlations over time (van de Schoot et al., 2012). Furthermore, most studies using ordered-categorical indicators (and all studies of *p*) did not test residual invariance (Bottesi et al., 2019; Deutz et al., 2016, 2018; Fong et al., 2022; Forbes et al., 2021; Gluschkoff et al., 2019; Grygiel et al., 2019; Hawes et al., 2018), which is required for change in latent means to be accurate when models are based on ordinal data (Liu et al., 2017; Millsap & Yun-Tein, 2004). We, therefore, reviewed simulation literature on MI testing relevant to bifactor models and applied our resulting recommendations using an empirical example.

Based on a review of the MI simulation literature, the following recommendations are made for MI cut-offs for bifactor models: (a) due to the complexity of bifactor models, Δχ^2^ is inappropriate for invariance testing, even more so when large samples are used (e.g., ≥1,000) (Cheung & Rensvold, 2002); (b) ΔCFI<0.01 appears to be an acceptable indicator of MI (all the way to residual invariance) for bifactor models with continuous and ordered-categorical indicators (e.g., F. F. Chen, 2007; Cheung & Rensvold, 2002; Sass et al., 2014); (c) ΔRMSEA<0.007 also appears to be an acceptable indicator of MI for models with ordered-categorical indicators in sample sizes of 1,000 or more (Sass et al., 2014), but ΔRMSEA is not recommended for MI testing in models with continuous indicators (Khojasteh & Lo, 2015; Meade et al., 2008). However, these guidelines must be applied with caution, as they are based on simulations of first-order models in all but one study (Khojasteh & Lo, 2015). This highlights the need for more simulations of MI testing in bifactor models, to devise appropriate cut-offs.

Additional caution is warranted for MI testing of models specified by many items. This is particularly relevant for structural psychopathology research, where models based on more comprehensive sets of symptoms are advocated to enable advancement in this field (Lahey et al., 2021). Furthermore, modeling a construct with too few indicators can hinder accurate detection of multidimensionality (Watts et al., 2021). To date, bifactor models of *p* have been much smaller than the 106 items from the empirical example, with most reviewed here ranging from 9 to 15 items (Caspi et al., 2014; Castellanos-Ryan et al., 2016; Class et al., 2019; Deutz et al., 2018; Forbes et al., 2021; Gluschkoff et al., 2019; Greene & Eaton, 2017; Lahey et al., 2012; Olino et al., 2018; Snyder et al., 2017). The concerns with larger models are as follows. First, there is a negative association between the number of items in a model and the incremental fit indices (e.g., CFI, TLI): models with 30+ indicators are more likely to yield problematic fit (Gignac, 2007). Second, ΔCFI and ΔRMSEA may not adequately detect parameter differences across groups in factors specified by many items, as noted in simulations of single-factor non-hierarchical models (D’Urso et al., 2021). Thus, inspection of parameter changes over time (or groups) is advisable in larger models, even if MI cut-offs support invariance. Such inspection revealed some non-invariance in the empirical example. However, the practical impact of this non-invariance is unknown, as simulations of various levels of non-invariance in bifactor models have yet to be performed (Nye et al., 2019).

The inability of ΔCFI and ΔRMSEA to detect non-invariance may be a function of model complexity independent of scale length, as some non-invariance was present in bifactor models of 11–15 items which met MI cut-offs (Deutz et al., 2018; Forbes et al., 2021; Gluschkoff et al., 2019). The complexity of the bifactor model, such as multiple latent dimensions, residual factors, and cross-loadings, makes it tend to fit any type of data well (Bonifay & Cai, 2017). The impact of this complexity of the ability to identify non-invariance must be addressed in future simulations. Such work should also jointly consider how the complexity and number of indicators in a model affect MI testing.

The present article underscores the challenges in estimator choice for MI testing of bifactor models specifically with ordered-categorical indicators: (a) the need for residual invariance testing in ordered-categorical data means that ML-based approaches are currently not appropriate; (b) all levels of MI testing are possible using WLSMV, but single-group longitudinal MI testing is computationally intensive for large bifactor models, and may not converge with multiple waves; (c) Bayesian estimation, a full-information approach which for large models has greater convergence rates than WLSMV (Garnier-Villarreal et al., 2021), still needs development for MI testing of polytomous indicators. Regarding indicator type, we note that convergence rates for MLR (assuming a continuous distribution) and robust categorical least squares (similar to WLSMV) increase as the number of categories increases (Rhemtulla et al., 2012). Thus, it is possible the convergence issues we observed would not have occurred if more categories were present in our data, or continuous indicators were used. Further work should explore these issues. In the meantime, simulations testing the appropriateness of the multigroup model for longitudinal MI are warranted.

More attention needs to be paid to the testing and reporting of heterotypic paths as part of longitudinal MI, as this was inconsistent in our literature review of longitudinal MI in bifactor models. To our knowledge, only bifactor models of psychopathology have reported heterotypic path coefficients, yet in models with varying degrees of longitudinal MI. Our empirical example revealed differing conclusions regarding heterotypic paths depending on the level of MI modeled. For an accurate understanding of continuities and discontinuities of general and specific factors over time, homotypic and heterotypic paths need to be reported at sufficient levels of MI testing, and compared across studies at equivalent levels of MI. These cross-wave covariances between latent factors are accurate when at least metric invariance has been established with continuous indicators, or scalar invariance with ordered-categorical indicators (Liu et al., 2017).

As missing data are common in longitudinal studies, we note the following regarding MI testing with missing data rates up to 50%: (a) when testing scalar invariance with ML in unbalanced groups due to attrition or otherwise, the ΔCFI and certainly ΔRMSEA cut-offs we advocate above appear to be acceptable (Yoon & Lai, 2018); (b) although a limited-information estimator, WLSMV appears to be acceptable for MI testing in ordered-categorical data in sample sizes of 1,000 or more (P.-Y. Chen et al., 2020). Alternatives to mitigate bias due to missingness using WLSMV are infeasible for large, complex models (adding predictors of missingness can be prohibitively computationally burdensome (Asparouhov & Múthen, 2010; B. O. Muthén et al., 2015) or in need of further development (pooling nested model test statistics across multiply imputed datasets is currently only possible for χ^2^, not CFI and RMSEA (Liu & Sriutaisuk, 2021).

## Conclusion

Our review and empirical example highlights the limitations in longitudinal MI testing of bifactor models. Nonetheless, using the guidelines advocated herein to establish MI allows findings from bifactor models to be more confidently interpreted. Such increased clarity will help improve comparability and consistency across the literature pertaining to these highly utilized models.

## Supplemental Material

sj-docx-1-asm-10.1177_10731911231182687 – Supplemental material for Measurement Invariance in Longitudinal Bifactor Models: Review and Application Based on the p Factor Supplemental material, sj-docx-1-asm-10.1177_10731911231182687 for Measurement Invariance in Longitudinal Bifactor Models: Review and Application Based on the p Factor by Sharon A. S. Neufeld, Michelle St Clair, Jeannette Brodbeck, Paul O. Wilkinson, Ian M. Goodyer and Peter B. Jones in Assessment

## References

[bibr1-10731911231182687] Agtarap S. Kramer M. D. Campbell-Sills L. Yuh E. Mukherjee P. Manley G. T. McCrea M. A. Dikmen S. Giacino J. T. Stein M. B. Nelson L. D. Adeoye O. Badjatia N. Boase K. Bodien Y. Bullock M. R. Chesnut R. Corrigan J. D. Crawford K. . . . Zafonte R. (2021). Invariance of the bifactor structure of Mild Traumatic Brain Injury (mTBI) symptoms on the rivermead postconcussion symptoms questionnaire across time, demographic characteristics, and clinical groups: A TRACK-TBI study. Assessment, 28(6), 1656–1670. 10.1177/1073191120913941 32326739 PMC7584771

[bibr2-10731911231182687] Asparouhov T. Múthen B. (2010). Weighted least squares estimation with missing data. https://www.statmodel.com/download/GstrucMissingRevision.pdf

[bibr3-10731911231182687] Bonifay W. Cai L. (2017). On the complexity of item response theory models. Multivariate Behavioral Research, 52(4), 465–484. 10.1080/00273171.2017.1309262 28426237

[bibr4-10731911231182687] Bottesi G. Noventa S. Freeston M. H. Ghisi M. (2019). Seeking certainty about intolerance of uncertainty: Addressing old and new issues through the intolerance of uncertainty scale-revised. PLOS ONE, 14(2), Article e0211929. 10.1371/journal.pone.0211929 PMC6370219 30742694

[bibr5-10731911231182687] Brett B. L. Kramer M. D. McCrea M. A. Broglio S. P. McAllister T. W. Nelson L. D. Hazzard J. B. Kelly L. A. Ortega J. Port N. Pasquina P. F. Jackson J. Cameron K. L. Houston M. N. Goldman J. T. Giza C. Buckley T. Clugston J. R. Schmidt J. D. . . . Susmarski A. (2020). Bifactor model of the sport concussion assessment tool symptom checklist: Replication and invariance across time in the CARE Consortium sample. American Journal of Sports Medicine, 48(11), 2783–2795. 10.1177/0363546520946056 32809856 PMC7484253

[bibr6-10731911231182687] Brown T. A. (2006). Confirmatory factor analysis for applied research. Guildford Press.

[bibr7-10731911231182687] Cai L. (2010). Metropolis-Hastings Robbins-Monro Algorithm for Confirmatory Item Factor Analysis. Journal of Educational and Behavioral Statistics, 35(3), 307–335. 10.3102/1076998609353115

[bibr8-10731911231182687] Caspi A. Houts R. M. Belsky D. W. Goldman-Mellor S. J. Harrington H. Israel S. Meier M. H. Ramrakha S. Shalev I. Poulton R. Moffitt T. E. (2014). The p factor: One general psychopathology factor in the structure of psychiatric disorders? Clinical Psychological Science, 2(2), 119–137. 10.1177/2167702613497473 25360393 PMC4209412

[bibr9-10731911231182687] Caspi A. Moffitt T. E. (2018). All for one and one for all: Mental disorders in one dimension. American Journal of Psychiatry, 175(9), 831–844. 10.1176/appi.ajp.2018.17121383 29621902 PMC6120790

[bibr10-10731911231182687] Castellanos-Ryan N. Briere F. N. O’Leary-Barrett M. Banaschewski T. Bokde A. Bromberg U. Biichel C. Flor H. Frouin V. Gallinat J. Garavan H. Martinot J. L. Nees F. Paus T. Pausova Z. Rietschel M. Smolka M. N. Robbins T. W. Whelan R. , . . . The IMAGEN Consortium. (2016). The structure of psychopathology in adolescence and its common personality and cognitive correlates. Journal of Abnormal Psychology, 125(8), 1039–1052. 10.1037/abn0000193 27819466 PMC5098414

[bibr11-10731911231182687] Chen F. F. (2007). Sensitivity of goodness of fit indexes to lack of measurement invariance. Structural Equation Modeling, 14(3), 464–504. 10.1080/10705510701301834

[bibr12-10731911231182687] Chen P.-Y. Wu W. Garnier-Villarreal M. Kite B. A. Jia F. (2020). Testing measurement invariance with ordinal missing data: A comparison of estimators and missing data techniques. Multivariate Behavioral Research, 55(1), 87–101. 10.1080/00273171.2019.1608799 31099262

[bibr13-10731911231182687] Cheung G. W. Rensvold R. B. (2002). Evaluating goodness-of-fit indexes for testing measurement invariance. Structural Equation Modeling, 9(2), 233–255. 10.1207/S15328007SEM0902

[bibr14-10731911231182687] Class Q. A. Van Hulle C. A. Rathouz P. J. Applegate B. Zald D. H. Lahey B. B. (2019). Socioemotional dispositions of children and adolescents predict general and specific second-order factors of psychopathology in early adulthood: A 12-year prospective study. Journal of Abnormal Psychology, 128(6), 574–584. 10.1037/abn0000433 31259570 PMC6786758

[bibr15-10731911231182687] D’Urso E. D. De Roover K. Vermunt J. K. Tijmstra J. (2021). Scale length does matter: Recommendations for measurement invariance testing with categorical factor analysis and item response theory approaches. Behavior Research Methods, 54, 2114–2145. 10.3758/s13428-021-01690-7 34910286 PMC9579096

[bibr16-10731911231182687] Deary I. J. (2012). Intelligence. Annual Review of Psychology, 63, 453–482. 10.1146/annurev-psych-120710-100353 21943169

[bibr17-10731911231182687] Deutz M. H. F. Geeraerts S. B. Belsky J. Deković M. van Baar A. L. Prinzie P. Patalay P. (2020). General psychopathology and dysregulation profile in a longitudinal community sample: Stability, antecedents and outcomes. Child Psychiatry & Human Development, 51(1), 114–126. 10.1007/s10578-019-00916-2 31359330

[bibr18-10731911231182687] Deutz M. H. F. Geeraerts S. B. van Baar A. L. Dekovice M. Prinzie P. (2016). The Dysregulation Profile in middle childhood and adolescence across reporters: Factor structure, measurement invariance, and links with self-harm and suicidal ideation. European Child & Adolescent Psychiatry, 25(4), 431–442. 10.1007/s00787-015-0745-x 26226917 PMC4820491

[bibr19-10731911231182687] Deutz M. H. F. Shi Q. Vossen H. G. M. Huijding J. Prinzie P. Deković M. van Baar A. L. Woltering S. (2018). Evaluation of the Strengths and Difficulties Questionnaire-Dysregulation Profile (SDQ-DP). Psychological Assessment, 30(9), 1174–1185. 10.1037/pas0000564 29927304 PMC6107405

[bibr20-10731911231182687] Fong T. C. T. Yip P. S. F. Ho R. T. H . (2022). Psychometric validation of the Chinese Health Questionnaire among young people in Hong Kong across 2018 and 2019. Psychological Assessment, 34(3), 261–270. 10.1037/pas0001079 34843284

[bibr21-10731911231182687] Forbes M. K. Greene A. L. Levin-Aspenson H. F. Watts A. L. Hallquist M. Lahey B. B. Markon K. E. Patrick C. J. Tackett J. L. Waldman I. D. Wright A. G. C. Caspi A. Ivanova M. Kotov R. Samuel D. B. Eaton N. R. Krueger R. F. (2021). Three recommendations based on a comparison of the reliability and validity of the predominant models used in research on the empirical structure of psychopathology. Journal of Abnormal Psychology, 130, 297–317. 10.1037/abn0000533 33539117

[bibr22-10731911231182687] Garnier-Villarreal M. Jorgensen T. D. (2020). Adapting fit indices for bayesian structural equation modeling: Comparison to maximum likelihood. Psychological Methods, 25(1), 46–70. 10.1037/met0000224 31180693

[bibr23-10731911231182687] Garnier-Villarreal M. Merkle E. C. Magnus B. E. (2021). Between-item multidimensional IRT: How far can the estimation methods go? Psych, 3, 404–421. 10.3390/psych3030029

[bibr24-10731911231182687] Gignac G. E. (2007). Multi-factor modeling in individual differences research: Some recommendations and suggestions. Personality and Individual Differences, 42(1), 37–48. 10.1016/j.paid.2006.06.019

[bibr25-10731911231182687] Gluschkoff K. Jokela M. Rosenström T. (2019). The general psychopathology factor: Structural stability and generalizability to within-individual changes. Frontiers in Psychiatry, 10, Article 594. 10.3389/fpsyt.2019.00594 PMC6728891 31543833

[bibr26-10731911231182687] Greene A. L. Eaton N. R. (2017). The temporal stability of the bifactor model of comorbidity: An examination of moderated continuity pathways. Comprehensive Psychiatry, 72, 74–82. 10.1016/j.comppsych.2016.09.010 27764677

[bibr27-10731911231182687] Grygiel P. Humenny G. Rębisz S. (2019). Using the De Jong Gierveld Loneliness Scale with early adolescents: Factor structure, reliability, stability, and external validity. Assessment, 26(2), 151–165. 10.1177/1073191116682298 27932403

[bibr28-10731911231182687] Guenole N. Brown A. (2014). The consequences of ignoring measurement invariance for path coefficients in structural equation models. Frontiers in Psychology, 5, Article 980. 10.3389/fpsyg.2014.00980 PMC4166111 25278911

[bibr29-10731911231182687] Harris P. A. Taylor R. Minor B. L. Elliott V. Fernandez M. O’Neal L. McLeod L. Delacqua G. Delacqua F. Kirby J. Duda S. N. (2019). The REDCap consortium: Building an international community of software platform partners. Journal of Biomedical Informatics, 95, 103208. 10.1016/j.jbi.2019.103208 31078660 PMC7254481

[bibr30-10731911231182687] Harris P. A. Taylor R. Thielke R. Payne J. Gonzalez N. Conde J. G. (2009). Research Electronic Data Capture (REDCap)—A metadata-driven methodology and workflow process for providing translational research informatics support. Journal of Biomedical Informatics, 42(2), 377–381. 10.1016/j.jbi.2008.08.010 18929686 PMC2700030

[bibr31-10731911231182687] Hawes S. W. Byrd A. L. Kelley S. E. Gonzalez R. Edens J. F. Pardini D. A. (2018). Psychopathic features across development: Assessing longitudinal invariance among Caucasian and African American youths. Journal of Research in Personality, 73, 180–188. 10.1016/j.jrp.2018.02.003 31937980 PMC6959475

[bibr32-10731911231182687] Joo SH. Kim E.S. (2019). Impact of error structure misspecification when testing measurement invariance and latent-factor mean difference using MIMIC and multiple-group confirmatory factor analysis. Behav Res 51, 2688–2699. 10.3758/s13428-018-1124-6 30242617

[bibr33-10731911231182687] Kessler R. C. Amminger G. P. Aguilar-Gaxiola S. Alonso J. Lee S. Ustün T. B. (2007). Age of onset of mental disorders: A review of recent literature. Current Opinion in Psychiatry, 20(4), 359–364. 10.1097/YCO.0b013e32816ebc8c 17551351 PMC1925038

[bibr34-10731911231182687] Khojasteh J. Lo W. J. (2015). Investigating the sensitivity of goodness-of-fit indices to detect measurement invariance in a bifactor model. Structural Equation Modeling, 22(4), 531–541. 10.1080/10705511.2014.937791

[bibr35-10731911231182687] Kiddle B. Inkster B. Prabhu G. Moutoussis M. Whitaker K. J. , the NSPN Consortium, Bullmore E. T. Dolan R. J. Fonagy P. Goodyer I. M. Jones P. B . (2018). Cohort profile: The NSPN 2400 Cohort: A developmental sample supporting the Wellcome Trust NeuroScience in Psychiatry Network. International Journal of Epidemiology, 47(1), 18–19g. 10.1093/ije/dyx117 PMC5837633 29177462

[bibr36-10731911231182687] Lahey B. B. Applegate B. Hakes J. K. Zald D. H. Hariri A. R. Rathouz P. J. (2012). Is there a general factor of prevalent psychopathology during adulthood? Journal of Abnormal Psychology, 121(4), 971–977. 10.1037/a0028355 22845652 PMC4134439

[bibr37-10731911231182687] Lahey B. B. Moore T. M. Kaczkurkin A. N. Zald D. H. (2021). Hierarchical models of psychopathology: Empirical support, implications, and remaining issues. World Psychiatry, 20(1), 57–63. 10.1002/wps.20824 33432749 PMC7801849

[bibr38-10731911231182687] Little T. D. (2013). Methodology in the social sciences. Longitudinal structural equation modeling. Guilford Press.

[bibr39-10731911231182687] Liu Y. Millsap R. E. West S. G. Tein J. Tanaka R. Grimm K. J. (2017). Testing measurement invariance in longitudinal data with ordered-categorical measures. Psychological Methods, 22(3), 486–506. 10.1037/met0000075 27213981 PMC5121102

[bibr40-10731911231182687] Liu Y. Sriutaisuk S. (2021). A comparison of FIML- versus Multiple-imputation-based methods to test measurement invariance with incomplete ordinal variables. Structural Equation Modeling, 28, 590–608. 10.1080/10705511.2021.1876520

[bibr41-10731911231182687] Markon K. E. (2019). Bifactor and hierarchical models: Specification, inference, and interpretation. Annual Review of Clinical Psychology, 15(1), 51–69. 10.1146/annurev-clinpsy-050718-095522 30649927

[bibr42-10731911231182687] McElroy E. Belsky J. Carragher N. Fearon P. Patalay P. (2018). Developmental stability of general and specific factors of psychopathology from early childhood to adolescence: Dynamic mutualism or p-differentiation? Journal of Child Psychology and Psychiatry and Allied Disciplines, 59(6), 667–675. 10.1111/jcpp.12849 29197107 PMC6001631

[bibr43-10731911231182687] Meade A. W. Johnson E. C. Braddy P. W. (2008). Power and sensitivity of alternative fit indices in tests of measurement invariance. Journal of Applied Psychology, 93(3), 568–592. 10.1037/0021-9010.93.3.568 18457487

[bibr44-10731911231182687] Millsap R. E. Yun-Tein J. (2004). Assessing factorial invariance in ordered categorial measures. Multivariate Behavioral Research, 39(3), 479–515. 10.1207/S15327906MBR3903_4

[bibr45-10731911231182687] Muthén B. O. Muthén L. K. Asparouhov T. (2015). Estimator choices with categorical outcomes. https://www.statmodel.com/download/EstimatorChoices.pdf

[bibr46-10731911231182687] Muthén L. K. Muthén B. O. (2017). Mplus user’s guide (8th ed.). https://www.statmodel.com/download/usersguide/MplusUserGuideVer_8.pdf

[bibr47-10731911231182687] Noordhof A. Krueger R. F. Ormel J. Oldehinkel A. J. Hartman C. A. (2015). Integrating autism-related symptoms into the dimensional internalizing and externalizing model of psychopathology. The TRAILS study. Journal of Abnormal Child Psychology, 43(3), 577–587. 10.1007/s10802-014-9923-4 25099360

[bibr48-10731911231182687] Nye C. D. Bradburn J. Olenick J. Bialko C. Drasgow F. (2019). How big are my effects? Examining the magnitude of effect sizes in studies of measurement equivalence. Organizational Research Methods, 22(3), 678–709. 10.1177/1094428118761122

[bibr49-10731911231182687] Olino T. M. Bufferd S. J. Dougherty L. R. Dyson M. W. Carlson G. A. Klein D. N. (2018). The development of latent dimensions of psychopathology across early childhood: Stability of dimensions and moderators of change. Journal of Abnormal Child Psychology, 46, 1373–1383. 10.1007/s10802-018-0398-6 29359267 PMC6056348

[bibr50-10731911231182687] Porsius J. T. Martens A. L. Slottje P. Claassen L. Korevaar J. C. Timmermans D. R. M. Vermeulen R. Smid T. (2015). Somatic symptom reports in the general population: Application of a bi-factor model to the analysis of change. Journal of Psychosomatic Research, 79(5), 378–383. 10.1016/j.jpsychores.2015.09.006 26526312

[bibr51-10731911231182687] Putnick D. L. Bornstein M. H. (2016). Measurement invariance conventions and reporting: The state of the art and future directions for psychological research. Developmental Review, 41, 71–90. 10.1016/j.dr.2016.06.004 27942093 PMC5145197

[bibr52-10731911231182687] Rhemtulla M. Brosseau-Liard P. É. Savalei V. (2012). When can categorical variables be treated as continuous? A comparison of robust continuous and categorical SEM estimation methods under suboptimal conditions. Psychological Methods, 17(3), 354–373. 10.1037/a0029315 22799625

[bibr53-10731911231182687] Rodriguez A. Reise S. P. Haviland M. G. (2016). Applying bifactor statistical indices in the evaluation of psychological measures. Journal of Personality Assessment, 98(3), 223–237. 10.1080/00223891.2015.1089249 26514921

[bibr54-10731911231182687] Sass D. A. Schmitt T. A. Marsh H. W. (2014). Evaluating model fit with ordered categorical data within a measurement invariance framework: A comparison of estimators. Structural Equation Modeling, 21(2), 167–180. 10.1080/10705511.2014.882658

[bibr55-10731911231182687] Schlechter P. Wilkinson P. O. Ford T. Neufeld S. (2023). The Short Mood and Feelings Questionnaire from adolescence to emerging adulthood: Measurement invariance across time and sex. Psychological Assessment, 35, 405–418. 36951690 10.1037/pas0001222

[bibr56-10731911231182687] Seddig D. Leitgöb H. (2018). Approximate measurement invariance and longitudinal confirmatory factor analysis: Concept and application with panel data. Survey Research Methods, 12(1), 29–41. 10.18148/srm/2018.v12i1.7210

[bibr57-10731911231182687] Snyder H. R. Young J. F. Hankin B. L. (2017). Strong homotypic continuity in common psychopathology-, internalizing-, and externalizing-specific factors over time in adolescents. Clinical Psychology Science, 5(1), 98–110. 10.1177/2167702616651076 PMC5320894 28239532

[bibr58-10731911231182687] Stark S. Chernyshenko O. S. Drasgow F. (2006). Detecting differential item functioning with confirmatory factor analysis and item response theory: Toward a unified strategy. Journal of Applied Psychology, 91(6), 1292–1306. 10.1037/0021-9010.91.6.1292 17100485

[bibr59-10731911231182687] St Clair M. C. Neufeld S. Jones P. B. Fonagy P. Bullmore E. T. Dolan R. J. Moutoussis M. Toseeb U. Goodyer I. M . (2017). Characterising the latent structure and organisation of self-reported thoughts, feelings and behaviours in adolescents and young adults. PLOS ONE, 12(4), Article e0175381. 10.1371/journal.pone.0175381 PMC5389661 28403164

[bibr60-10731911231182687] van de Schoot R. Lugtig P. Hox J. (2012). A checklist for testing measurement invariance. European Journal of Developmental Psychology, 9(4), 486–492. 10.1080/17405629.2012.686740

[bibr61-10731911231182687] Vandenberg R. J. Lance C. E. (2000). A review and synthesis of the measurement invariance literature: Suggestions, practices, and recommendations for organizational research. Organizational Research Methods, 3(1), 4–69. 10.1177/109442810031002

[bibr62-10731911231182687] Watts A. L. Boness C. L. Loeffelman J. E. Steinley D. Sher K. J . (2021). Does crude measurement contribute to observed unidimensionality of psychological constructs? A demonstration with DSM–5 alcohol use disorder. Journal of Abnormal Psychology, 130(5), 512–524. 10.1037/abn0000678 34472887 PMC8443156

[bibr63-10731911231182687] Watts A. L. Lane S. P. Bonifay W. Steinley D. Meyer F. A. C. (2020). Building theories on top of, and not independent of, statistical models: The case of the p-factor. Psychological Inquiry, 31(4), 310–320. https://doi.org/doi:10.1080/1047840x.2020.1853476 33510565 10.1080/1047840x.2020.1853476 PMC7839945

[bibr64-10731911231182687] Wu H. Estabrook R. (2016). Identification of confirmatory factor analysis models of different levels of invariance for ordered categorical outcomes. Psychometrika, 81(4), 1014–1045. 10.1007/s11336-016-9506-0 27402166 PMC5458787

[bibr65-10731911231182687] Yoon M. Lai M. H. C. (2018). Testing factorial invariance with unbalanced samples. Structural Equation Modeling, 25(2), 201–213. 10.1080/10705511.2017.1387859

[bibr66-10731911231182687] Yuan K. H. Chan W. (2016). Measurement invariance via multigroup SEM: Issues and solutions with chi-square-difference tests. Psychological Methods, 21(3), 405–426. 10.1037/met0000080 27266799

[bibr67-10731911231182687] Zhong X. Yuan K. H. (2011). Bias and efficiency in structural equation modeling: Maximum likelihood versus robust methods. Multivariate Behavioral Research, 46(2), 229–265. 10.1080/00273171.2011.558736 26741329

