# Supplementary material for: Measurement Invariance in Longitudinal Bifactor Models: Review and Application Based on the p Factor
Source: Assessment. 2023 Jun 22;31(4):774–93. doi: 10.1177/10731911231182687 (PMC11092300; doi:10.1177/10731911231182687)
Supplement: sj-docx-1-asm-10.1177_10731911231182687 – Supplemental material for Measurement Invariance in Longitudinal Bifactor Models: Review and Application Based on the p Factor [file sj-docx-1-asm-10.1177_10731911231182687.docx]

**Supplementary Materials: Measurement invariance in longitudinal bifactor models: review and application based on the *p* factor**

**Supplement 1: Empirical Example – Results**

**Initial bifactor modelling**:

As more participants were recruited after developing the initial bifactor model,^1^ the baseline model was re-run with these additional participants. This resulted in a model with nearly identical fit statistics to those originally reported (Table S2), with all loadings significant and above cut-offs (≥0.30 for the general factor and ≥0.15 for specific factors). Five modification indices >100 indicated additional loadings onto specific factors. The theoretically appropriate loading with the highest index was added to the model. However, as the chi-square did not improve by 10% in an already good-fitting model,^2^ it was not necessary to make this or further modifications.

**Sparse data**

Consistent with the baseline model, ﻿responses on the Antisocial Behaviour Questionnaire were recoded to a binary response (“never” vs. “sometimes/mostly/always”) due to sparse endorsement of the latter two categories.^1^ Following this recoding, the number of response categories across all waves was consistent, so constraining thresholds was straightforward.^3^ Only eight participants endorsed ABQ11 (“I have deliberately hurt or been cruel to an animal”) at wave 3, which resulted in empty cells when correlated with two other items. Such sparsity could lead to inaccurate model estimation.^4^ Note that this item also had a loading below the 0.30 cut-off on the general factor in females (0.24). As the item in question was already dichotomized, there were no response categories which could be collapsed. Therefore, in order to assess the potential influence of this sparsity in the data, sensitivity analysis was performed on longitudinal invariance models and the wave 3 model by dropping this item^5^ and comparing model fit with and without this item.

Given comparable MI findings but with over 6 weeks more computation time using the single-group approach (see below), sensitivity analyses of the effects of sparse data were performed using the multigroup approach. Model fit for multigroup invariance testing models and the wave 3 model remained excellent without this item (Table S2). Without ABQ11, ΔCFI and ΔRMSEA differed by no more than 0.001 for MI models, and thus invariance conclusions were not altered. Further, using generated factor scores at wave 3, all factor autocorrelations with and without this item were 1.00. Together, these findings support minimal impact of ABQ11 sparsity in wave 3 of the model, and therefore inclusion of this item in the model is warranted. We note this item showed a medium-to-large amount of non-invariance on loadings (waves 2-3: Δλ_general_=0.276 Δλ_specific_=0.207; waves 1-3: Δλ_general_=0.270, Δλ_specific_=0.223) and a small amount of threshold non-invariance (waves 1-3: Δτ=0.309; all non-invariance levels based on Nye et al, 2018^6^). However, dropping this item does not materially change the amount of non-invariance on relevant factors.

**Computation time**

Due to lack of convergence for the single-group model with all three waves tested simultaneously, this model had to be broken down into three dyadic models (waves 1-2, waves 2-3, waves 1-3) and each model took at minimum nearly 90 hours to run (Table S2). Thus, across all levels of invariance testing, this resulted in a total of 1061 hours and 33 minutes (44 days, 5.6 hours) of computation time for the single-group MI testing. Conversely, across all levels of MI testing, the multigroup model took a total of 29 hours and 33 minutes (1 day, 5.4 hours) of computation time, approximately 43 days less computation time than the single-group model. These computation times are an underestimate of the actual computation required, as they are only reflective of the final models. Additional computation time was required for the single-group models to assess the need for longitudinal cross-factor correlations and residual correlations. Some models also needed to be re-run with additional iterations to ensure convergence, and random starts to replicate the best fit function several times.

**Table S1**: Original measures, constructs, and item loadings from the empirical example of the bifactor model of psychopathology (*p*)^a^

| **Self-report measure,^b^ number of items (code preface for items)** | **Key constructs assessed** | **Item response set** | **Number of items loading onto each factor** | | | | | | |
| --- | --- | --- | --- | --- | --- | --- | --- | --- | --- |
|  |  |  | ***p* (general factor)** | **sf1^c^**  **Self-confidence** | **sf2 Anti-social** | **sf3 Worry** | **sf4 Aberrant thoughts** | **sf5**  **Mood** | **sf positive worded items^d^** |
| Warwick-Edinburgh Mental Well-Being Scale (WEMWBS),^7^ 14 items (wb_01-14) | Mental well-being: evidence of current happiness, personal activity and personal achievement | 0-4 | 13 (inverse) | 13 |  |  |  | 8  (inverse) | 13 |
| Antisocial Behaviour Questionnaire (ABQ),^e^ 11 items (ypq_73-83) | antisocial behaviour: violating social norms, destructive behaviours, violence to people, lying and stealing | 0-1 | 8 |  | 8 |  |  |  |  |
| Revised Children's Manifest Anxiety Scale (RCMAS),^8^ 28 items (ypq_34-61) | Generalized anxiety: physiological change, worry / oversensitivity, social concerns | 0-3, | 25 |  |  | 7 |  | 1^f^ |  |
| Revised Leyton Obsessional Inventory (LOI),^9^ 11 items (ypq_62-72) | Obsessions and compulsions | 0-3 | 11 |  |  |  | 8 |  |  |
| Schizotypal Personality Questionnaire (SPQ),^10^ 11 items related to psychotic-like experiences^g^ (spq_) | total symptoms, hallucinations, delusions, and unusual perceptual experiences | 0-1 | 10 |  |  |  | 9 |  |  |
| Rosenberg Self-Esteem Scale (RSES),^11^ 10 items (ypq_84-93) | self-esteem | 0-3 | 10  (5 inverse) |  |  |  |  | 9  (5 inverse) | 5 |
| Moods and Feelings Questionnaire (MFQ),^12^ 33 items (ypq_01-33) | depression | 0-3, | 29 |  |  |  |  | 12 |  |
| **TOTAL ITEMS** |  |  | **106** | **13** | **8** | **7** | **17** | **30** | **18** |

^a^ model is based on St Clair et al, 2017,^1^ with MFQ24 dropped from the antisocial specific factor as done for the gender-invariant model (see results section for details). All items loaded on *p*.

^b^ for all measures, participants were asked to rate their thoughts, behaviours, and emotions over the past two weeks, for all but SPQ (see below). Of the original 118 items, 12 items were removed due to very sparse endorsement or low loading on *p* (<0.30^1^)

^c^ sf=specific factor, named to reflect the content of item loadings^1^

^d^ all positively worded items specified this method factor

^e^ developed for use in a prior adolescent cohort study^13^. Items were recoded from 0-3 to binary due to sparse endorsement.^1^

^f^ item is “Other people my age were happier than me”

^g^ SPQ items exhibiting a highly significant relationship (medium to large effect size) and face validity with psychotic-like experiences on the semi-structured PLIKS interview (PLIKSi^14^) from participants the In-Unit-Assessment subsample. See St Clair et al, 2017 for details.

**Table S2**: Factor loadings for the bifactor model, separate by wave

| **item** | **Wording** | **Wave 1** | | | | | | | **Wave 2** | | | | | | | **Wave 3** | | | | | | |
| --- | --- | --- | --- | --- | --- | --- | --- | --- | --- | --- | --- | --- | --- | --- | --- | --- | --- | --- | --- | --- | --- | --- |
|  |  | ***p*-factor** | **sf1** | **sf2** | **sf3** | **sf4** | **sf5** | **sf pos** | ***p*-factor** | **sf1** | **sf2** | **sf3** | **sf4** | **sf5** | **sf pos** | ***p*-factor** | **sf1** | **sf2** | **sf3** | **sf4** | **sf5** | **sf pos** |
| MFQ1 | Felt miserable or unhappy | 0.704 |  |  |  |  | 0.256 |  | 0.729 |  |  |  |  | 0.261 |  | 0.715 |  |  |  |  | 0.272 |  |
| MFQ2 | Didn't enjoy anything | 0.622 |  |  |  |  | 0.247 |  | 0.701 |  |  |  |  | 0.232 |  | 0.724 |  |  |  |  | 0.302 |  |
| MFQ5 | So tired/sat around and did nothing | 0.540 |  |  |  |  |  |  | 0.561 |  |  |  |  |  |  | 0.564 |  |  |  |  |  |  |
| MFQ6 | Moving and walking more slowly than usual | 0.540 |  |  |  |  |  |  | 0.590 |  |  |  |  |  |  | 0.517 |  |  |  |  |  |  |
| MFQ7 | Was very restless | 0.489 |  |  |  |  |  |  | 0.552 |  |  |  |  |  |  | 0.565 |  |  |  |  |  |  |
| MFQ8 | Felt I was no good any more | 0.791 |  |  |  |  | 0.345 |  | 0.820 |  |  |  |  | 0.312 |  | 0.845 |  |  |  |  | 0.296 |  |
| MFQ9 | Sometimes blamed myself for things not my fault | 0.711 |  |  |  |  |  |  | 0.741 |  |  |  |  |  |  | 0.755 |  |  |  |  |  |  |
| MFQ11 | Got grumpy and cross easily | 0.606 |  |  |  |  |  |  | 0.651 |  |  |  |  |  |  | 0.681 |  |  |  |  |  |  |
| MFQ12 | Felt like talking less than usual | 0.643 |  |  |  |  |  |  | 0.662 |  |  |  |  |  |  | 0.692 |  |  |  |  |  |  |
| MFQ13 | Talking more slowly than usual | 0.567 |  |  |  |  |  |  | 0.643 |  |  |  |  |  |  | 0.537 |  |  |  |  |  |  |
| MFQ14 | Cried a lot | 0.648 |  |  |  |  |  |  | 0.636 |  |  |  |  |  |  | 0.707 |  |  |  |  |  |  |
| MFQ15 | Thought there was nothing good for me in future | 0.738 |  |  |  |  | 0.386 |  | 0.776 |  |  |  |  | 0.394 |  | 0.774 |  |  |  |  | 0.373 |  |
| MFQ16 | Thought life was not worth living | 0.791 |  |  |  |  | 0.336 |  | 0.760 |  |  |  |  | 0.368 |  | 0.786 |  |  |  |  | 0.386 |  |
| MFQ17 | Thought about dying | 0.695 |  |  |  |  |  |  | 0.705 |  |  |  |  |  |  | 0.722 |  |  |  |  |  |  |
| MFQ18 | Thought my family would be better off without me | 0.740 |  |  |  |  | 0.216 |  | 0.753 |  |  |  |  | 0.245 |  | 0.810 |  |  |  |  | 0.275 |  |
| MFQ19 | Thought about killing myself | 0.754 |  |  |  |  | 0.250 |  | 0.748 |  |  |  |  | 0.278 |  | 0.747 |  |  |  |  | 0.248 |  |
| MFQ20 | Didn't want to see friends | 0.694 |  |  |  |  |  |  | 0.737 |  |  |  |  |  |  | 0.712 |  |  |  |  |  |  |
| MFQ21 | Found it hard to think properly or concentrate | 0.739 |  |  |  |  |  |  | 0.779 |  |  |  |  |  |  | 0.777 |  |  |  |  |  |  |
| MFQ22 | Thought bad things would happen to me | 0.767 |  |  |  |  |  |  | 0.777 |  |  |  |  |  |  | 0.807 |  |  |  |  |  |  |
| MFQ23 | Hated myself | 0.828 |  |  |  |  | 0.328 |  | 0.823 |  |  |  |  | 0.317 |  | 0.863 |  |  |  |  | 0.309 |  |
| MFQ24 | I was a bad person | 0.746 |  |  |  |  |  |  | 0.770 |  |  |  |  |  |  | 0.801 |  |  |  |  |  |  |
| MFQ25 | Thought I looked ugly | 0.644 |  |  |  |  | 0.313 |  | 0.714 |  |  |  |  | 0.192 |  | 0.705 |  |  |  |  | 0.166 |  |
| MFQ26 | Worried about aches and pains | 0.470 |  |  |  |  |  |  | 0.491 |  |  |  |  |  |  | 0.493 |  |  |  |  |  |  |
| MFQ27 | Felt lonely | 0.705 |  |  |  |  | 0.248 |  | 0.754 |  |  |  |  | 0.192 |  | 0.731 |  |  |  |  | 0.220 |  |
| MFQ28 | Thought nobody really loved me | 0.763 |  |  |  |  | 0.283 |  | 0.803 |  |  |  |  | 0.244 |  | 0.823 |  |  |  |  | 0.346 |  |
| MFQ29 | Didn't have any fun at school/work | 0.629 |  |  |  |  |  |  | 0.669 |  |  |  |  |  |  | 0.654 |  |  |  |  |  |  |
| MFQ30 | Thought I could never been as good as others | 0.768 |  |  |  |  | 0.245 |  | 0.789 |  |  |  |  | 0.231 |  | 0.784 |  |  |  |  | 0.265 |  |
| MFQ31 | I did everything wrong | 0.837 |  |  |  |  |  |  | 0.855 |  |  |  |  |  |  | 0.877 |  |  |  |  |  |  |
| MFQ32 | Didn’t sleep as well as usual | 0.532 |  |  |  |  |  |  | 0.573 |  |  |  |  |  |  | 0.604 |  |  |  |  |  |  |
| RCMAS1 | Had trouble making up my mind | 0.607 |  |  |  |  |  |  | 0.679 |  |  |  |  |  |  | 0.707 |  |  |  |  |  |  |
| RCMAS2 | Worried when things did not go the right way for me | 0.710 |  |  | 0.254 |  |  |  | 0.777 |  |  | 0.243 |  |  |  | 0.792 |  |  | 0.231 |  |  |  |
| RCMAS3 | Others seem to do things more easily than me | 0.765 |  |  |  |  |  |  | 0.800 |  |  |  |  |  |  | 0.835 |  |  |  |  |  |  |
| RCMAS4 | Trouble getting breath | 0.567 |  |  |  |  |  |  | 0.617 |  |  |  |  |  |  | 0.604 |  |  |  |  |  |  |
| RCMAS5 | Worried a lot of the time | 0.783 |  |  | 0.486 |  |  |  | 0.797 |  |  | 0.481 |  |  |  | 0.824 |  |  | 0.432 |  |  |  |
| RCMAS6 | Afraid of a lot of things | 0.787 |  |  | 0.353 |  |  |  | 0.809 |  |  | 0.306 |  |  |  | 0.815 |  |  | 0.323 |  |  |  |
| RCMAS7 | Got angry easily | 0.640 |  |  |  |  |  |  | 0.68 |  |  |  |  |  |  | 0.731 |  |  |  |  |  |  |
| RCMAS8 | Worried about what my parents would say | 0.628 |  |  |  |  |  |  | 0.673 |  |  |  |  |  |  | 0.710 |  |  |  |  |  |  |
| RCMAS9 | Felt that others did not like the way I did things | 0.733 |  |  |  |  |  |  | 0.792 |  |  |  |  |  |  | 0.777 |  |  |  |  |  |  |
| RCMAS10 | Hard for me to get to sleep at night | 0.549 |  |  |  |  |  |  | 0.633 |  |  |  |  |  |  | 0.573 |  |  |  |  |  |  |
| RCMAS11 | Worried about what others thought about me | 0.747 |  |  |  |  |  |  | 0.791 |  |  |  |  |  |  | 0.798 |  |  |  |  |  |  |
| RCMAS12 | Felt alone even when there were people with me | 0.810 |  |  |  |  |  |  | 0.850 |  |  |  |  |  |  | 0.863 |  |  |  |  |  |  |
| RCMAS13 | Often felt sick to my stomach | 0.692 |  |  |  |  |  |  | 0.741 |  |  |  |  |  |  | 0.738 |  |  |  |  |  |  |
| RCMAS16 | I was tired a lot | 0.624 |  |  |  |  |  |  | 0.669 |  |  |  |  |  |  | 0.699 |  |  |  |  |  |  |
| RCMAS17 | Worried about what was going to happen | 0.781 |  |  | 0.353 |  |  |  | 0.798 |  |  | 0.378 |  |  |  | 0.812 |  |  | 0.369 |  |  |  |
| RCMAS18 | Other people my age were happier than me | 0.799 |  |  |  |  | 0.219 |  | 0.833 |  |  |  |  | 0.208 |  | 0.829 |  |  |  |  | 0.219 |  |
| RCMAS19 | Had bad dreams | 0.546 |  |  |  |  |  |  | 0.598 |  |  |  |  |  |  | 0.556 |  |  |  |  |  |  |
| RCMAS20 | Feelings got hurt when I was fussed at | 0.756 |  |  |  |  |  |  | 0.764 |  |  |  |  |  |  | 0.777 |  |  |  |  |  |  |
| RCMAS21 | Felt someone would tell me I did things the wrong way | 0.706 |  |  |  |  |  |  | 0.769 |  |  |  |  |  |  | 0.769 |  |  |  |  |  |  |
| RCMAS22 | Wake up scared some of the time | 0.633 |  |  |  |  |  |  | 0.746 |  |  |  |  |  |  | 0.733 |  |  |  |  |  |  |
| RCMAS23 | Worried when I went to bed at night | 0.677 |  |  | 0.304 |  |  |  | 0.754 |  |  | 0.235 |  |  |  | 0.728 |  |  | 0.333 |  |  |  |
| RCMAS25 | Wiggled in my seat a lot | 0.484 |  |  |  |  |  |  | 0.587 |  |  |  |  |  |  | 0.561 |  |  |  |  |  |  |
| RCMAS26 | Worried | 0.771 |  |  | 0.512 |  |  |  | 0.791 |  |  | 0.486 |  |  |  | 0.800 |  |  | 0.471 |  |  |  |
| RCMAS27 | A lot of people were against me | 0.764 |  |  |  |  |  |  | 0.809 |  |  |  |  |  |  | 0.827 |  |  |  |  |  |  |
| RCMAS28 | Worried about something bad happening to me | 0.744 |  |  | 0.24 |  |  |  | 0.794 |  |  | 0.225 |  |  |  | 0.786 |  |  | 0.298 |  |  |  |
| LOI1 | Had to do things in a certain way to stop bad things happening | 0.547 |  |  |  | 0.518 |  |  | 0.588 |  |  |  | 0.650 |  |  | 0.463 |  |  |  | 0.738 |  |  |
| LOI2 | Trouble finishing things as I had to do things over and over again | 0.589 |  |  |  |  |  |  | 0.634 |  |  |  |  |  |  | 0.627 |  |  |  |  |  |  |
| LOI3 | Hated dirt and dirty things | 0.351 |  |  |  | 0.264 |  |  | 0.442 |  |  |  | 0.292 |  |  | 0.433 |  |  |  | 0.280 |  |  |
| LOI4 | Special number to count to or did things a certain number of times | 0.392 |  |  |  | 0.607 |  |  | 0.481 |  |  |  | 0.680 |  |  | 0.443 |  |  |  | 0.715 |  |  |
| LOI5 | Felt guilty about things even though no one thought I'd done anything wrong | 0.719 |  |  |  |  |  |  | 0.769 |  |  |  |  |  |  | 0.788 |  |  |  |  |  |  |
| LOI6 | Worried about being clean enough | 0.486 |  |  |  | 0.278 |  |  | 0.509 |  |  |  | 0.282 |  |  | 0.566 |  |  |  | 0.318 |  |  |
| LOI7 | Moved or talked in a special way to avoid bad luck | 0.378 |  |  |  | 0.653 |  |  | 0.493 |  |  |  | 0.683 |  |  | 0.367 |  |  |  | 0.732 |  |  |
| LOI8 | Worried if I did something not exactly how I liked | 0.605 |  |  |  | 0.356 |  |  | 0.671 |  |  |  | 0.423 |  |  | 0.661 |  |  |  | 0.436 |  |  |
| LOI9 | Fussy about keeping my hands clean | 0.343 |  |  |  | 0.288 |  |  | 0.402 |  |  |  | 0.315 |  |  | 0.439 |  |  |  | 0.323 |  |  |
| LOI10 | had a special number or words that I said to keep bad luck away | 0.424 |  |  |  | 0.719 |  |  | 0.468 |  |  |  | 0.762 |  |  | 0.503 |  |  |  | 0.767 |  |  |
| LOI11 | Thinking about things as wasn't sure they had been done the right way | 0.716 |  |  |  |  |  |  | 0.729 |  |  |  |  |  |  | 0.714 |  |  |  |  |  |  |
| ABQ1 | Deliberately broke rules or disobeyed people | 0.432 |  | 0.657 |  |  |  |  | 0.473 |  | 0.668 |  |  |  |  | 0.477 |  | 0.658 |  |  |  |  |
| ABQ2 | Stole things | 0.377 |  | 0.664 |  |  |  |  | 0.420 |  | 0.608 |  |  |  |  | 0.339 |  | 0.763 |  |  |  |  |
| ABQ3 | Deliberately damaged property | 0.346 |  | 0.707 |  |  |  |  | 0.394 |  | 0.708 |  |  |  |  | 0.427 |  | 0.758 |  |  |  |  |
| ABQ4 | Deliberately hurt or threatened someone | 0.398 |  | 0.625 |  |  |  |  | 0.382 |  | 0.705 |  |  |  |  | 0.415 |  | 0.705 |  |  |  |  |
| ABQ5 | Skipped lessons/work or played truant for school/work | 0.342 |  | 0.462 |  |  |  |  | 0.381 |  | 0.410 |  |  |  |  | 0.431 |  | 0.573 |  |  |  |  |
| ABQ6 | Deliberately lied or cheated to get what I want | 0.434 |  | 0.599 |  |  |  |  | 0.383 |  | 0.645 |  |  |  |  | 0.402 |  | 0.695 |  |  |  |  |
| ABQ7 | Ran away from home | 0.525 |  | 0.480 |  |  |  |  | 0.590 |  | 0.434 |  |  |  |  | 0.591 |  | 0.661 |  |  |  |  |
| ABQ11 | Deliberately hurt or been cruel to an animal | 0.335 |  | 0.535 |  |  |  |  | 0.329 |  | 0.551 |  |  |  |  | 0.605 |  | 0.758 |  |  |  |  |
| RSE1 | At times, thought I was no good at all | 0.830 |  |  |  |  | 0.341 |  | 0.840 |  |  |  |  | 0.323 |  | 0.854 |  |  |  |  | 0.311 |  |
| RSE2+ | Was satisfied with myself | -0.603 |  |  |  |  | -0.355 | 0.485 | -0.605 |  |  |  |  | -0.358 | 0.521 | -0.591 |  |  |  |  | -0.323 | 0.546 |
| RSE3+ | Felt I had a number of good qualities | -0.541 |  |  |  |  | -0.373 | 0.604 | -0.550 |  |  |  |  | -0.409 | 0.611 | -0.567 |  |  |  |  | -0.362 | 0.644 |
| RSE4+ | Was able to do things as well as most people | -0.567 |  |  |  |  | -0.309 | 0.549 | -0.601 |  |  |  |  | -0.283 | 0.597 | -0.629 |  |  |  |  | -0.292 | 0.595 |
| RSE5 | Felt I did not have much to be proud of | 0.717 |  |  |  |  | 0.407 |  | 0.736 |  |  |  |  | 0.401 |  | 0.727 |  |  |  |  | 0.396 |  |
| RSE6 | Certainly felt useless at times | 0.798 |  |  |  |  | 0.334 |  | 0.816 |  |  |  |  | 0.293 |  | 0.788 |  |  |  |  | 0.295 |  |
| RSE7+ | Felt that I was as good as anyone else | -0.549 |  |  |  |  | -0.307 | 0.495 | -0.550 |  |  |  |  | -0.334 | 0.538 | -0.527 |  |  |  |  | -0.333 | 0.513 |
| RSE8 | Wished I could have more respect for myself | 0.626 |  |  |  |  |  |  | 0.656 |  |  |  |  |  |  | 0.677 |  |  |  |  |  |  |
| RSE9 | Felt I was a failure | 0.805 |  |  |  |  | 0.387 |  | 0.823 |  |  |  |  | 0.348 |  | 0.839 |  |  |  |  | 0.357 |  |
| RSE10+ | I took a positive attitude towards myself | -0.602 |  |  |  |  | -0.448 | 0.479 | -0.642 |  |  |  |  | -0.379 | 0.491 | -0.631 |  |  |  |  | -0.402 | 0.490 |
| SPQ4 | Often mistaken objects for people or noises for voices? | 0.377 |  |  |  | 0.399 |  |  | 0.429 |  |  |  | 0.393 |  |  | 0.432 |  |  |  | 0.340 |  |  |
| SPQ9 | I am sure I am being talked about behind my back | 0.593 |  |  |  |  |  |  | 0.675 |  |  |  |  |  |  | 0.654 |  |  |  |  |  |  |
| SPQ13 | Had the sense of a person/force is around you, even if you can't see it? | 0.336 |  |  |  | 0.490 |  |  | 0.370 |  |  |  | 0.472 |  |  | 0.357 |  |  |  | 0.528 |  |  |
| SPQ28 | Noticed a common event/object that seemed to be a special sign for you? | 0.343 |  |  |  | 0.473 |  |  | 0.322 |  |  |  | 0.487 |  |  | 0.375 |  |  |  | 0.515 |  |  |
| SPQ31 | Often hear a voice speaking my thoughts aloud. | 0.330 |  |  |  | 0.400 |  |  | 0.424 |  |  |  | 0.269 |  |  | 0.363 |  |  |  | 0.384 |  |  |
| SPQ40 | Seen things invisible to other people? | 0.353 |  |  |  | 0.522 |  |  | 0.524 |  |  |  | 0.482 |  |  | 0.390 |  |  |  | 0.584 |  |  |
| SPQ60 | Sometimes feel that other people are watching you? | 0.534 |  |  |  | 0.355 |  |  | 0.555 |  |  |  | 0.302 |  |  | 0.608 |  |  |  | 0.356 |  |  |
| SPQ61 | Suddenly feel distracted by distant sounds you're not normally aware of? | 0.407 |  |  |  | 0.492 |  |  | 0.495 |  |  |  | 0.454 |  |  | 0.477 |  |  |  | 0.383 |  |  |
| SPQ63 | Sometimes feel that people are talking about you? | 0.529 |  |  |  | 0.207 |  |  | 0.571 |  |  |  | 0.216 |  |  | 0.592 |  |  |  | 0.237 |  |  |
| SPQ64 | Thoughts sometimes so strong you can almost hear them? | 0.440 |  |  |  | 0.456 |  |  | 0.532 |  |  |  | 0.357 |  |  | 0.534 |  |  |  | 0.398 |  |  |
| WB1+ | Feeling optimistic about future | -0.466 | 0.305 |  |  |  | -0.471 | 0.197 | -0.508 | 0.366 |  |  |  | -0.405 | 0.163 | -0.546 | 0.379 |  |  |  | -0.424 | 0.207 |
| WB2+ | Feeling useful | -0.512 | 0.327 |  |  |  | -0.370 | 0.231 | -0.601 | 0.365 |  |  |  | -0.326 | 0.168 | -0.600 | 0.397 |  |  |  | -0.327 | 0.230 |
| WB3+ | Feeling relaxed | -0.575 | 0.420 |  |  |  |  | 0.195 | -0.626 | 0.423 |  |  |  |  | 0.206 | -0.628 | 0.441 |  |  |  |  | 0.215 |
| WB5+ | Had energy to spare | -0.397 | 0.389 |  |  |  |  | 0.216 | -0.460 | 0.433 |  |  |  |  | 0.206 | -0.511 | 0.448 |  |  |  |  | 0.200 |
| WB6+ | Been dealing with problems well | -0.578 | 0.422 |  |  |  |  | 0.347 | -0.640 | 0.413 |  |  |  |  | 0.306 | -0.646 | 0.456 |  |  |  |  | 0.330 |
| WB7+ | Been thinking clearly | -0.625 | 0.449 |  |  |  |  | 0.321 | -0.679 | 0.406 |  |  |  |  | 0.337 | -0.685 | 0.455 |  |  |  |  | 0.289 |
| WB8+ | Feeling good about myself | -0.649 | 0.387 |  |  |  | -0.407 | 0.290 | -0.718 | 0.407 |  |  |  | -0.320 | 0.276 | -0.715 | 0.421 |  |  |  | -0.315 | 0.266 |
| WB9+ | Feeling close to other people | -0.437 | 0.474 |  |  |  | -0.324 | 0.055 | -0.512 | 0.475 |  |  |  | -0.282 | 0.098 | -0.515 | 0.472 |  |  |  | -0.348 | 0.108 |
| WB10+ | Feeling confident | -0.561 | 0.427 |  |  |  | -0.380 | 0.292 | -0.646 | 0.429 |  |  |  | -0.308 | 0.235 | -0.673 | 0.446 |  |  |  | -0.297 | 0.281 |
| WB11+ | Been able to make up my own mind about things | -0.525 | 0.334 |  |  |  |  | 0.310 | -0.607 | 0.347 |  |  |  |  | 0.280 | -0.593 | 0.380 |  |  |  |  | 0.275 |
| WB12+ | Feeling loved | -0.502 | 0.409 |  |  |  | -0.349 | 0.047 | -0.532 | 0.410 |  |  |  | -0.337 | 0.121 | -0.613 | 0.386 |  |  |  | -0.398 | 0.074 |
| WB13+ | Been interested in new things | -0.360 | 0.416 |  |  |  | -0.253 | 0.155 | -0.454 | 0.447 |  |  |  | -0.220 | 0.228 | -0.467 | 0.522 |  |  |  | -0.276 | 0.166 |
| WB14+ | Been feeling cheerful | -0.627 | 0.481 |  |  |  | -0.326 | 0.166 | -0.676 | 0.499 |  |  |  | -0.312 | 0.152 | -0.665 | 0.491 |  |  |  | -0.352 | 0.185 |

*p*-factor=general psychopathology factor, sf=specific factor. See Table S1 for full names of measures. WB=WEMWBS.

All loadings significant p<.001, except 2 items loading onto “sf pos”: at wave 1, WB9 (p<.01) and WB12 (p<.05); at wave 3, WB12 (p<.01). Loading cut-offs not considered for this methods factor.

+=positively worded items, load onto “sf pos” factor

**Table S3:** Fit indices and computation time for measurement invariance testing of the bifactor model of psychopathology (*p*) from the empirical example

| **Model** | **n** | **Chi Square (χ^2^)** | **df** | **# of free parameters** | **CFI** | **TLI** | **RMSEA** | **Computation time^a^** (hours:minutes:seconds) |
| --- | --- | --- | --- | --- | --- | --- | --- | --- |
| Baseline only models |  |  |  |  |  |  |  |  |
| Original model^b^ (St Clair et al, 2017) | 2228 | 15859 | 5350 | 510 | 0.956 | 0.955 | 0.030 | --- |
| Original model final data | 2372 | 16718 | 5350 | 510 | 0.955 | 0.954 | 0.030 | --- |
| Waves 1&2 invariance single-group | 2389 |  |  |  |  |  |  |  |
| configural |  | 35951 | 21933 | 1023 | 0.964 | 0.963 | 0.016 | 89:36:39 |
| scalar |  | 36063 | 22307 | 649 | 0.965 | 0.964 | 0.016 | 142:56:40 |
| residual |  | 34506 | 22413 | 543 | 0.969 | 0.969 | 0.015 | 113:31:16 |
| Waves 2&3 invariance single-group | 1687 |  |  |  |  |  |  |  |
| configural |  | 29998 | 21933 | 1023 | 0.974 | 0.973 | 0.015 | 110:44:42 |
| scalar |  | 30166 | 22307 | 649 | 0.974 | 0.974 | 0.014 | 124:55:02 |
| residual |  | 29498 | 22413 | 543 | 0.977 | 0.977 | 0.014 | 132:11:26 |
| Waves 1&3 invariance single-group | 2382 |  |  |  |  |  |  |  |
| configural |  | 30373 | 21933 | 1023 | 0.975 | 0.974 | 0.013 | 140:52:19 |
| scalar |  | 30599 | 22307 | 649 | 0.975 | 0.975 | 0.012 | 92:26:38 |
| residual |  | 30144 | 22413 | 543 | 0.977 | 0.977 | 0.012 | 114:18:24 |
| Waves 1&3 invariance single-group, heterotypic paths | 2382 |  |  |  |  |  |  |  |
| configural |  | 30747 | 21891 | 1065 | 0.974 | 0.973 | 0.013 | 105:51:08 |
| scalar |  | 30916 | 22265 | 692 | 0.974 | 0.974 | 0.013 | 98:25:55 |
| residual |  | 30316 | 22371 | 585 | 0.976 | 0.976 | 0.012 | 101:47:31 |
| Waves 1-3 invariance multigroup | 5127 |  |  |  |  |  |  |  |
| configural |  | 36471 | 16083 | 1497 | 0.967 | 0.966 | 0.027 | 21:23:58 |
| scalar |  | 35876 | 16808 | 772 | 0.969 | 0.970 | 0.026 | 05:05:44 |
| residual |  | 29201 | 17050 | 530 | 0.980 | 0.981 | 0.020 | 02:55:14 |
| As above, without ABQ11 (empty cells) |  |  |  |  |  |  |  |  |
| Wave 3 model | 1096 | 9368 | 5248 | 506 | 0.972 | 0.971 | 0.027 |  |
| Waves 1-3 multigroup invariance | 5127 |  |  |  |  |  |  | --- |
| configural |  | 37006 | 15774 | 1488 | 0.966 | 0.964 | 0.028 | --- |
| scalar |  | 36238 | 16495 | 767 | 0.968 | 0.968 | 0.026 | --- |
| residual |  | 28981 | 16735 | 527 | 0.980 | 0.981 | 0.021 | --- |

^a^ for comparison of final single-group and multigroup models only

^b^ MFQ24 (“I was a bad person”) loaded on the antisocial behaviour specific factor in the original model. This was dropped (but retained on the general factor) for all subsequent models due to a low loading in females.

**Table S4**: Heterotypic and homotypic paths from the bifactor model of psychopathology (*p*) from the empirical example

|  | ***p* (general factor) T3** | **self-confidence (sf1) T3** | **antisocial (sf2) T3** | **worry (sf3) T3** | **aberrant thoughts (sf4) T3** | **mood (sf5) T3** | **positive methods sf T3** |
| --- | --- | --- | --- | --- | --- | --- | --- |
| ***p* (general factor) T1** | .697*** / .618*** | .079** / .045 | -.126^+^ / -.098^+^ | -.063 / -.051 | .099* / .100** | -.073^+^ / -.068^+^ | -.079^+^ / -.074^+^ |
| **self-confidence (sf1) T1** | . 059 / .071^+^ | .415*** / .398*** | .058 / .045 | -.036 / -.035 | .058 / .055 | .026 / .021 | .083^+^ / .064 |
| **antisocial (sf2) T1** | .038 / .030 | -.048 / -.045 | .861*** / .726*** | -.276*** / -.264*** | .099 / .090 | .016 / .024 | .160^+^ / .132^+^ |
| **worry (sf3) T1** | -.034 / -.036 | -.012 / -.011 | -.222** / -.193** | .591*** / .579*** | .025 / .023 | -.004 / -.009 | -.138* / -.118^+^ |
| **aberrant thoughts (sf4) T1** | .099* / .081* | .037 / .035 | .226** / .195** | -.004 / -.003 | .736*** / .660*** | .096^+^ / .097^+^ | .004 / .002 |
| **mood (sf5) T1** | -.068^+^ / -.059^+^ | .104** / .116*** | .008 / .007 | -.056 / -.061 | .070 / .062 | .502*** / .503*** | .142*** / .123*** |
| **positive methods sf T1** | -.094* / -.083** | .043 / .055 | .114 / .097 | -.064 / -.063 | .068 / .060 | .025 / .022 | .424*** / .382*** |

*p*=general factor of psychopathology; T=timepoint; sf=specific factor

^+^ p<.05; *p<.01; **p<=.005; ***p<=.001. Effect sizes:^15^ 0.10–0.29 (small), 0.30–0.49 (medium), 0.50+ (large)

Note: Each cell contains two standardized estimates, under conditions of scalar and residual measurement invariance (MI), respectively. Since at least scalar invariance is required for assessing covariances in ordered-categorical data,^3^ either estimate is acceptable to use if that level of MI is met. Both estimates are presented to illustrate any consequences of presenting results at differing levels of invariance. Although the scalar and residual heterotypic models are equivalent according to the strictest MI cut-offs (Table S2: ΔCFI did not worsen by .002 or more), the above shows the path from *p* T1 to self-confidence specific factor was only significant in the scalar invariant model. This effect size change was tiny (.034) across these levels of invariance, yet the precision of the effect was also much greater in the scalar invariant model, leading to different conclusions regarding significant heterotypic paths, depending on which level of MI was applied. The maximal effect size difference across the two levels of invariance was small (.135), observed in the homotypic effect with antisocial specific factor. However, as both effects were strong, this difference does not lead to a substantiative alteration in conclusions.

**References**

1. St Clair MC, Neufeld S, Jones PB, Fonagy P, Bullmore ET, Dolan RJ, et al. Characterising the latent structure and organisation of self-reported thoughts, feelings and behaviours in adolescents and young adults. PLoS One. 2017;12(4):e0175381.

2. Little TD. Methodology in the social sciences. Longitudinal structural equation modeling. New York, NY, US: Guilford Press; 2013.

3. Liu Y, Millsap RE, West SG, Tein J, Tanaka R, Grimm KJ. Testing measurement invariance in longitudinal data with ordered-categorical measures. Psychol Methods. 2017;22(3):486–506.

4. Flora DB, Curran PJ. An empirical evaluation of alternative methods of estimation for confirmatory factor analysis with ordinal data. Psychol Methods. 2004;9(4):466–91.

5. Muthén BO, Muthén LK, Asparouhov T. Estimator choices with categorical outcomes [Internet]. 2015. Available from: https://www.statmodel.com/download/ EstimatorChoices.pdf

6. Nye CD, Bradburn J, Olenick J, Bialko C, Drasgow F. How Big Are My Effects? Examining the Magnitude of Effect Sizes in Studies of Measurement Equivalence. Organ Res Methods. 2019;22(3):678–709.

7. Tennant R, Hiller L, Fishwick R, Platt S, Joseph S, Weich S, et al. The Warwick-Dinburgh mental well-being scale (WEMWBS): Development and UK validation. Health Qual Life Outcomes. 2007;5:1–13.

8. Reynolds C, Richmond B. What I think and feel: a revised measure of children’s manifest anxiety. J Abnorm Child Psychol. 1978;6(2):271–80.

9. Bamber D, Tamplin A, Park RJ, Kyte ZA, Goodyer IM. Development of a Short Leyton Obsessional Inventory for Children and Adolescents. J Am Acad Child Adolesc Psychiatry. 2002;41(10):1246–52.

10. Raine A. The SPQ: A scale for the assessment of schizotypal personality based on DSM-III-R criteria. Schizophr Bull. 1991;17(4):555–64.

11. Rosenberg M. Society and the adolescent self-image. Princeton, N.J.: Princeton University Press; 1965.

12. Costello EJ, Angold A. Scales to assess child and adolescent depression – checklists, screens, and nets. J Am Acad Child Psychiatry. 1988;27:726–737.

13. Goodyer IM, Croudace T, Dunn V, Herbert J, Jones PB. Cohort Profile: Risk patterns and processes for psychopathology emerging during adolescence: the ROOTS project. Int J Epidemiol. 2010;39(2):361–9.

14. Horwood J, Salvi G, Thomas K, Duffy L, Gunnell D, Hollis C, et al. IQ and non-clinical psychotic symptoms in 12-year-olds: Results from the ALSPAC birth cohort. Br J Psychiatry. 2008;193(3):185–91.

15. Cohen J. Statistical Power Analysis for the Behavioral Sciences. New York, NY: Routledge Academic; 1988.
